# Causes of death in rural southeast Asia by electronic verbal autopsy: a population-based observational study

**DOI:** 10.1016/S2214-109X(25)00362-6

**Published:** 2025-12-01

**Authors:** Nan Shwe Nwe Htun, Koukeo Phommasone, Carlo Perrone, Aung Pyae Phyo, Aninda Sen, Moul Vanna, Rupam Tripura, Nawrin Kabir, Md Akramul Islam, Jindaporn Wirachonphaophong, Patcharaporn Panyadee, Arjun Chandna, Supachai Boonyoung, Dysoley Lek, Tiengkham Pongvongsa, Mayfong Mayxay, Rajeev Kamadod, Nicholas P J Day, Shaun K Morris, Elizabeth A Ashley, Prabhat Jha, Sue J Lee, Yoel Lubell, Thomas J Peto

**Affiliations:** Mahidol Oxford Tropical Medicine Research Unit, Faculty of Tropical Medicine, Mahidol University, Bangkok, Thailand; Centre for Tropical Medicine and Global Health, Nuffield Department of Medicine, University of Oxford, Oxford, UK; Lao-Oxford-Mahosot Hospital-Wellcome Trust Research Unit, Microbiology Laboratory, Mahosot Hospital, Vientiane, Laos; Centre for Tropical Medicine and Global Health, Nuffield Department of Medicine, University of Oxford, Oxford, UK; Centre for Tropical Medicine and Global Health, Nuffield Department of Medicine, University of Oxford, Oxford, UK; Shoklo Malaria Research Unit, Mahidol-Oxford Tropical Medicine Research Unit, Faculty of Tropical Medicine, Mahidol University, Mae Ramat, Thailand; Communicable Diseases Programme, BRAC, Dhaka, Bangladesh; Action for Health Development, Battambang, Cambodia; Mahidol Oxford Tropical Medicine Research Unit, Faculty of Tropical Medicine, Mahidol University, Bangkok, Thailand; Communicable Diseases Programme, BRAC, Dhaka, Bangladesh; Shoklo Malaria Research Unit, Mahidol-Oxford Tropical Medicine Research Unit, Faculty of Tropical Medicine, Mahidol University, Mae Ramat, Thailand; Mahidol Oxford Tropical Medicine Research Unit, Faculty of Tropical Medicine, Mahidol University, Bangkok, Thailand; Centre for Tropical Medicine and Global Health, Nuffield Department of Medicine, University of Oxford, Oxford, UK; Cambodia Oxford Medical Research Unit, Angkor Hospital for Children, Siem Reap, Cambodia; Centre for Tropical Medicine and Global Health, Nuffield Department of Medicine, University of Oxford, Oxford, UK; Chiangrai Prachanukroh Hospital, Chiangrai, Thailand; National Center for Parasitology, Entomology, and Malaria Control, Phnom Penh, Cambodia; School of Public Health, National Institute of Public Health, Phnom Penh, Cambodia; Public Health Office, Savannakhet, Laos; Centre for Tropical Medicine and Global Health, Nuffield Department of Medicine, University of Oxford, Oxford, UK; Lao-Oxford-Mahosot Hospital-Wellcome Trust Research Unit, Microbiology Laboratory, Mahosot Hospital, Vientiane, Laos; Institute of Research and Education Development, University of Health Sciences, Ministry of Health, Vientiane, Laos; Saw Swee Hock School of Public Health, National University of Singapore, Singapore; Nuffield Department of Population Health, University of Oxford, Oxford, UK; Mahidol Oxford Tropical Medicine Research Unit, Faculty of Tropical Medicine, Mahidol University, Bangkok, Thailand; Centre for Tropical Medicine and Global Health, Nuffield Department of Medicine, University of Oxford, Oxford, UK; Division of Infectious Diseases, Child Health Evaluation Sciences and Centre for Global Child Health, Hospital for Sick Children, Toronto, ON, Canada; Faculty of Medicine and Dalla Lana School of Public Health, University of Toronto, Toronto, ON, Canada; Centre for Tropical Medicine and Global Health, Nuffield Department of Medicine, University of Oxford, Oxford, UK; Lao-Oxford-Mahosot Hospital-Wellcome Trust Research Unit, Microbiology Laboratory, Mahosot Hospital, Vientiane, Laos; Nuffield Department of Population Health, University of Oxford, Oxford, UK; Faculty of Medicine and Dalla Lana School of Public Health, University of Toronto, Toronto, ON, Canada; Mahidol Oxford Tropical Medicine Research Unit, Faculty of Tropical Medicine, Mahidol University, Bangkok, Thailand; Department of Infectious Diseases, The Alfred Hospital and School of Translational Medicine, Monash University, Melbourne, VIC, Australia; Mahidol Oxford Tropical Medicine Research Unit, Faculty of Tropical Medicine, Mahidol University, Bangkok, Thailand; Centre for Tropical Medicine and Global Health, Nuffield Department of Medicine, University of Oxford, Oxford, UK

## Abstract

**Background:**

In low-income and middle-income countries in southeast Asia, most deaths occur outside of the health-care system without a medically certified cause of death. We did a verbal autopsy study to determine the underlying causes of death in rural areas of the region and to estimate premature mortality using years of life lost (YLLs).

**Methods:**

In this population-based observational study, we conducted electronic verbal autopsy surveys for reported deaths in 510 villages in Bangladesh, Cambodia, Laos, Myanmar, and Thailand from 2021 to 2024. With the WHO 2016 verbal autopsy questionnaire, trained fieldworkers conducted interviews, and local physicians independently assigned causes of death by ICD-10 codes. Causes of death were ranked based on cause-specific mortality fractions (CSMFs), and YLLs calculated using corresponding national life expectancy.

**Findings:**

Over 90% of reported deaths had a verbal autopsy administered. Among 3413 deaths, 2052 (60%) were male and 1361 (40%) were female, and 3245 (95%) were older than 12 years. Across sites, 64–86% of deaths occurred at home. Non-communicable diseases were the leading causes of death, ranging from 54% in Laos to 70% in Cambodia, with cardiovascular, cerebrovascular, cancer, and digestive conditions being the most common. Maternal, nutritional, and communicable diseases, mainly respiratory infections, diarrhoea, and tuberculosis, accounted for 11–26% of deaths across the sites. Accidents and injuries comprised 7–13% and ranked among the top five causes. Major gaps in death documentation persist across all countries, whereas disease-specific causes of death varied between sites. Non-communicable diseases accounted for the majority of YLLs, representing between 56% and 65% across the five countries.

**Interpretation:**

This multicountry study highlighted the significant burden of non-communicable diseases in rural southeast Asia, alongside persistent communicable diseases, emphasising the need for better mortality data and health-care access to reduce this dual burden.

**Funding:**

Wellcome Trust.

## Introduction

Every year, millions of deaths go unregistered or do not have reliable data on the causes of death, creating crucial gaps in the evidence needed to set global health priorities. Accurate mortality data are fundamental for public health planning, both locally and internationally, yet most low-income and middle-income countries (LMICs) struggle with incomplete or unreliable death registration systems. Southeast Asia, home to around 700 million people, still faces significant challenges in capturing comprehensive mortality data due to logistical, systemic, and cultural barriers.^1^ According to WHO, only 69% of deaths in the region were registered, and only 17% had a medically certified cause in 2020.^2^ This issue is more pronounced in rural areas, where 65% of southeast Asia’s population still resides.^3^ In these settings, 40–80% of deaths occur at home and often go unreported.^4^ Even when deaths are recorded, the reliability of cause-of-death data remains questionable.^5^ Verbal autopsies—structured interviews with family members or caregivers to determine probable cause of death—have emerged as a practical and scalable solution in settings with weak registration systems or where medical certification is unavailable, providing cause-specific mortality estimates.^6^ Studies conducted in India, Ethiopia, Tanzania, Kenya, and Sierra Leone have shown that verbal autopsies, despite being less informative for those aged 70 years or older, can generate plausible cause-of-death estimates for early childhood and adult deaths.^7–10^ These studies also showed that verbal autopsy methods did well for broad cause-of-death categories and are most reliable when assigning causes at the population level.

The South and Southeast Asian Community-based Trials Network (SEACTN) is a research programme initiated in 2020 to investigate the aetiology and burden of rural febrile illnesses covering parts of five countries— Bangladesh, Myanmar, Laos, Thailand, and Cambodia.^11^ WHO has categorised these countries as having either poor or unusable cause-of-death data due to quality concerns, particularly in rural areas.^12^ Therefore, SEACTN incorporated a verbal autopsy study to estimate the proportion of deaths caused by febrile illnesses, and to understand causes of mortality in these populations more broadly. Additionally, we estimated cause-specific mortality fractions (CSMFs), premature mortality using years of life lost (YLLs), access to care during the final illness, and the completeness of local death reporting.

## Methods

### Study design and participants

To build trust and ensure cultural appropriateness, extensive public engagement activities were conducted before the study across all sites to understand local traditions and sensitivities surrounding death that could influence the study implementation.^13^ The study was done from Dec 1, 2021, to April 30, 2024 (the exact timing of data collection varied by site due to country-specific COVID-19 regulations). It covered 510 purposively selected rural villages in five countries: Bangladesh (Cox’s Bazar and Bandarban districts), Cambodia (Battambang and Pailin provinces), Laos (Phalanxay, Phine, and Atsaphanthong districts in Savannakhet Province), Myanmar (Hpa-An, Myawaddy, and Hlaing-Bwe districts in Karen State), and Thailand (Chiang Rai Province). Study villages were chosen based on their inclusion in the SEACTN rural febrile illness network (appendix 7 p 2).^11^ These villages were located in contiguous catchment areas linked to primary care units that do not have advanced diagnostic capabilities and specialist care. The study aimed to capture all reported deaths within the selected villages using multiple notification sources (appendix 7 p 2). An interview by a trained fieldworker was attempted for every reported death with predefined criteria^14^ to select the most suitable respondents, preferably close family members, caregivers, or individuals who attended to the deceased to ensure accuracy and reliability. Eligible respondents were adults (aged ≥18 years) whose family member had died in the study villages within the past 12 months.

The study received approvals from the Oxford Tropical Research Ethics Committee (OxTREC reference: 553–20) and national ethics committees in each participating country: BRAC James P Grant School of Public Health, Bangladesh (IRB-31 December’21–050); National Ethics Committees for Health Research in Laos (06/NECHR) and Cambodia (314/NECHR); Chiang Rai Public Health Province Ethics Committee, Thailand (HCR20007); and the Karen Department of Welfare and Health in Myanmar (approval dated Sept 23, 2022). Before interviews, study procedures were explained and written informed consent was obtained from all participants. Respondents who agreed to participate were compensated in culturally appropriate ways, such as household essentials, per local ethical guidelines. This trial is registered with ClinicalTrials.gov (NCT04595656).

### Procedures

A WHO 2016 electronic verbal autopsy questionnaire^14^ was used for collecting data for neonates (aged <29 days), children (aged 29 days to 12 years), and adolescents and adults (aged >12 years). These covered the deceased’s demographic information, medical history, symptoms (including pregnancy-related), injury history, risk factors, health-care usage, and the presence of medical and death certificates. A half-page local language narrative was included to describe symptom progression and treatment history in the respondent’s own words. Questionnaires were professionally translated into the local language for each site, and causes of death were classified using the ICD-10 coding system. Additionally, any official records of the death were collected where available; a death certificate is a physician-signed document stating the cause, date, and place of death, while death registration is a formal recording of a death at a local registrar office.

Five field teams (one per study site) and a total of 47 interviewers were trained using the WHO manual^14^ through online and in-person sessions in local languages, covering study objectives, questionnaire walkthroughs, role-play exercises, and the Open Data Kit platform. While the study field teams were responsible for conducting the interviews, three physicians per site were recruited to independently review and assign the underlying cause of death based on the interview records. They were trained via a web-based system by the Million Death Study group^15^ following WHO guidelines and ICD-10 coding standards, and passed assessments on the physician coding manual. A single underlying cause of death was reported, considering risk factors but not relying solely on them. With local stakeholder support, the research team approached families with care and sensitivity, and interviews were scheduled based on family preferences and cultural practices. Responses were entered into electronic tablets. To mitigate the risk of COVID-19 transmission during fieldwork, the study team followed prevention protocols including personal protective equipment use, physical distancing, and conducting interviews in well ventilated settings.

To ensure data quality, field supervisors observed a randomly selected 10% of interviews, to address issues affecting accuracy of the information collected. For cause-of-death determination, a double-coding system was implemented. Two local physicians independently coded each case, with disagreements undergoing reconciliation. Where differences persisted, a third physician adjudicated the case through a masked process, either selected one of the original causes of death or determined a new one. The WHO 2016 verbal autopsy instrument used in this study, which did not include COVID-19-specific questions, was supplemented with symptom-based criteria consistent with WHO clinical case definitions, and contextual information to identify probable COVID-19 deaths. Uploaded responses and coding data were checked by field teams and a study coordinator (NSNH) for duplications, errors, and missing details. Any inconsistencies in interview responses were reviewed thoroughly and, when necessary, verified with participants to maintain completeness and accuracy.

### Statistical analysis

Descriptive statistics included counts and proportions to summarise demographic data such as age, sex, place of death, health-care-seeking behaviour during the deceased’s final illness, types of health-care facilities accessed, and practices surrounding death documentation. Causes of death were categorised into four broad groups: non-communicable diseases (NCDs); communicable, maternal, perinatal, and nutritional conditions; accidents and injuries; and non-specific causes of death (symptoms, signs, and ill-defined conditions).^16^ Senility, defined as age-related mental and physical decline in individuals aged 70 years and older, was included under ill-defined conditions but reported separately to describe its distribution. Crude death rates were calculated as total deaths divided by the total study population, expressed per 1000 people. CSMFs were determined by dividing the number of deaths from a specific cause by the total number of verbal autopsies, expressed as percentages with 95% CIs adjusted for clustering by village by multiplying the standard error with a variance inflation factor. Although diseases were coded into individual ICD codes (eg, ischaemic heart disease), they were reported as broader categories (eg, cardiovascular conditions), which produce more reliable estimates than disease-specific subcategories. The groupings followed the verbal autopsy studies in Sierra Leone (appendix 7 p 3).^7^ YLLs for each country were calculated by multiplying deaths in each 5-year age group by the remaining life expectancy at the median age for each group at country level (appendix 7 pp 9–10),^17^ using binomial exact 95% CIs. The sum of all age groups provided the total YLLs, which was divided by study population and expressed per 100 000 population. This represents a crude, population-based YLL rate.

### Role of the funding source

The funder of the study had no role in study design, data collection, data analysis, data interpretation, or writing of the report.

## Results

**Figure 1:**
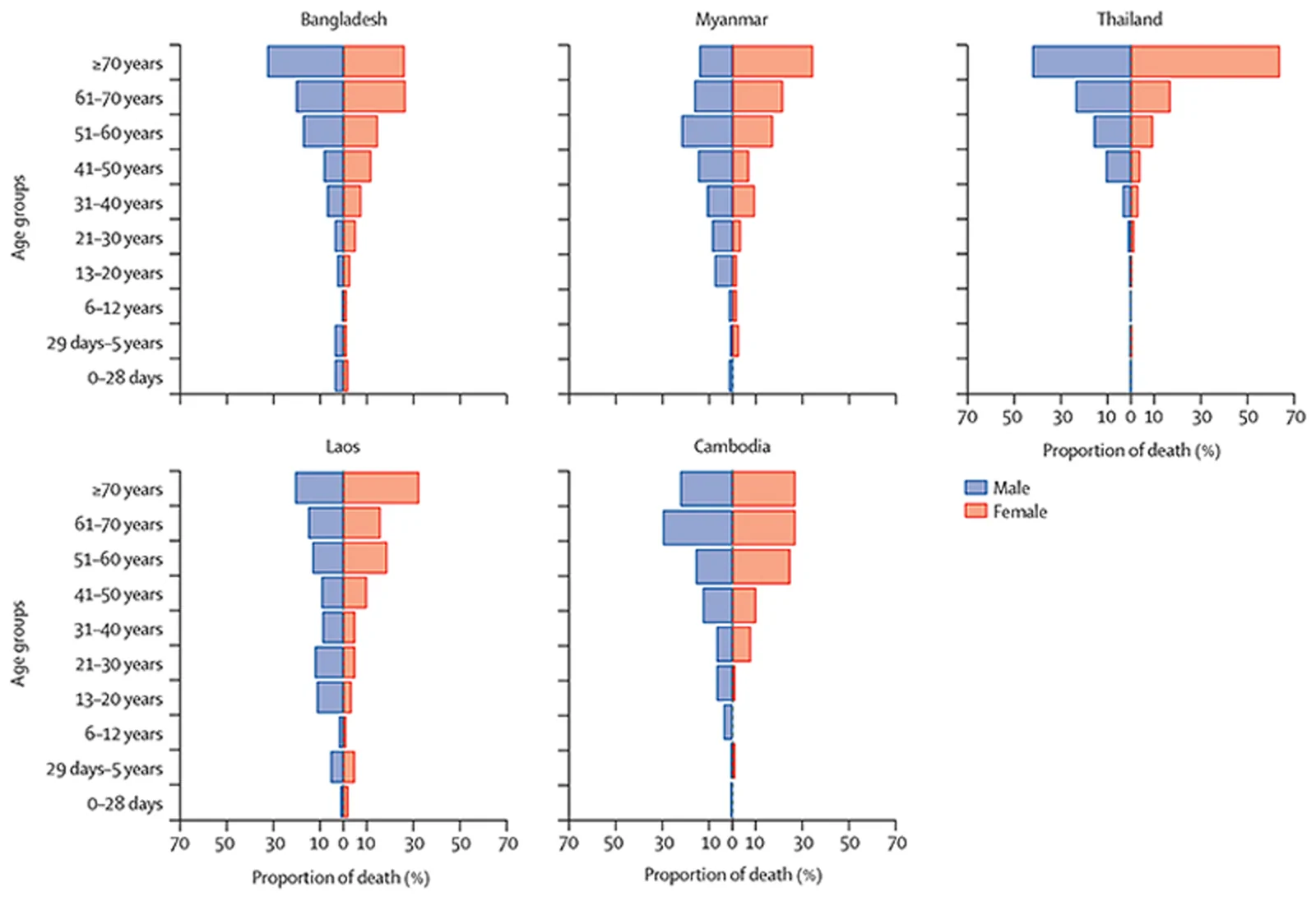
Age and sex distribution of deaths across study areas

Overall, 3733 deaths were reported and 3413 (91%) verbal autopsies were carried out (table 1). The majority (3245 [95%]) of verbal autopsies were of adult deaths and 1492 (44%) of the cases were from Laos, attributable to its larger catchment population. Across all countries, males accounted for approximately 60% (n=2052) of deaths. Except in Bangladesh and Laos, male deaths predominated in middle-aged adults (aged 40–70 years; figure 1), whereas among those aged 70 years or older, female deaths tended to exceed male deaths, especially in Thailand (>60%). Children and neonatal deaths accounted for a small proportion of total deaths compared with adults, representing 6% in Bangladesh, 3% in Myanmar, 7% in Laos, 1% in Cambodia, and less than 1% in Thailand. The majority of deaths (65% to 87% by country) occurred at home. Thailand had the highest percentage of deaths occurring in health facilities (31%), whereas Cambodia (18%) and Laos (8%) reported the lowest. The interview timing varied with mourning customs.^13^ Most interviews were conducted within 6 months of death in Bangladesh (98%), Cambodia (79%), and Myanmar (69%), whereas a higher proportion occurred between 6 months and 12 months in Thailand (39%) and Laos (44%). Thailand had the highest death certificate issuance (97%), followed by Cambodia (69%). In contrast, Laos had a very low rate (0·4%), and in Myanmar there were no deaths with an available death certificate or registration.

**Figure 2:**
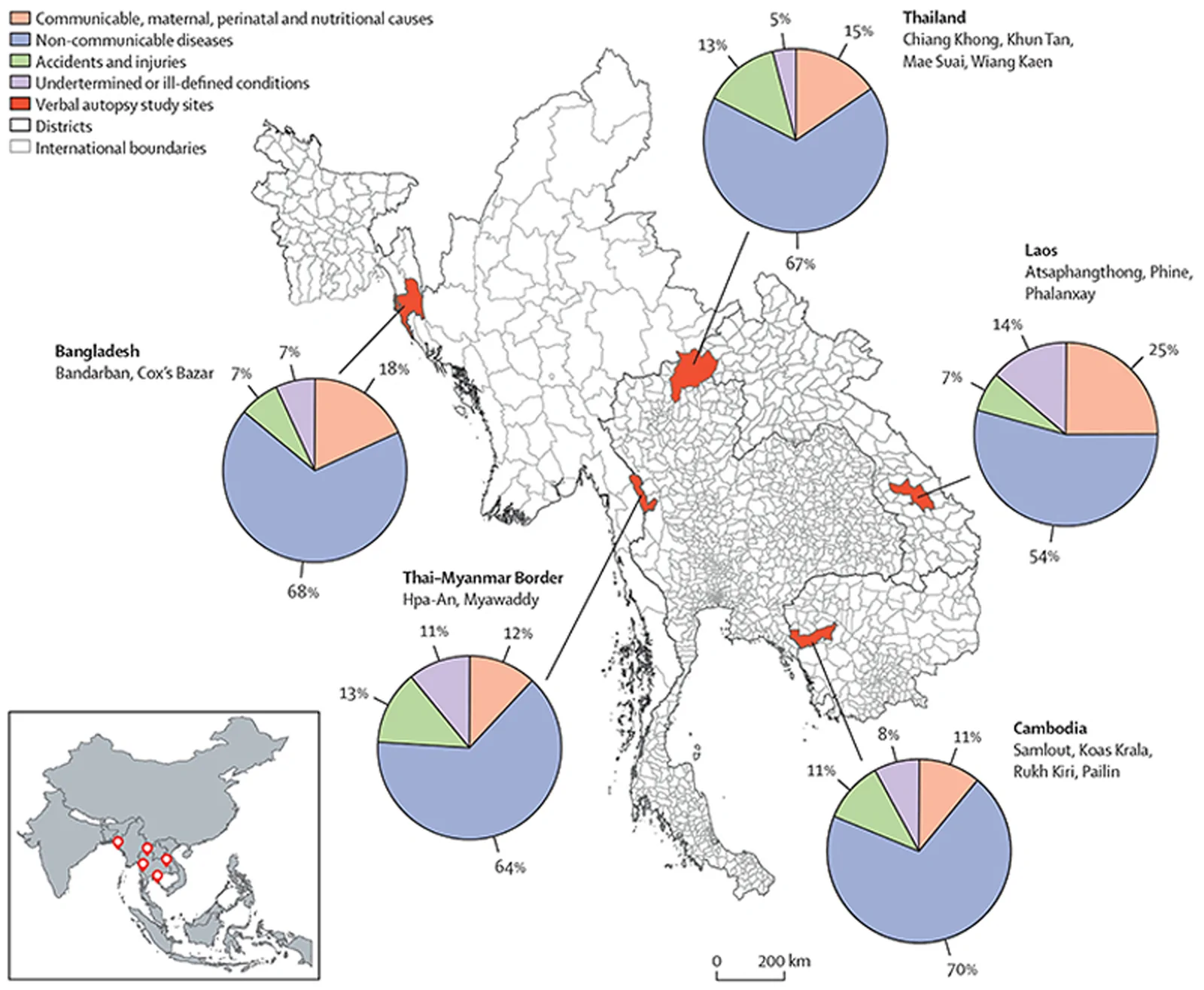
Study sites with proportion of deaths by major cause groups

Among the four major cause groups, NCDs were the leading cause of death across all sites, ranging from 54% in Laos to 70% in Cambodia (figure 2; appendix 7 p 11). Laos and Bangladesh had the highest proportions of deaths due to communicable, maternal, perinatal and nutritional conditions (25% and 18%, respectively). Accidents and injuries accounted for 7–13% of deaths across all sites. Under symptoms, signs, and ill-defined conditions, cases attributed to unknown cause of death (3%) were fewer compared with those classified as senility, which was more frequent in females in Laos, Cambodia, and Myanmar. Laos reported higher numbers of both senility and ill-defined conditions, especially among males than the other countries. In the distribution of age and sex, a higher proportion of older females was observed compared with males in Cambodia, Laos, and Myanmar (appendix 7 p 11).

We also report on the top ten causes of death according to CSMFs (table 2; appendix 7 p 11). Cardiovascular diseases (eg, ischaemic heart diseases, rheumatic heart diseases, and hypertensive heart diseases) were in the top five causes in Bangladesh, Thailand, Laos, and Cambodia, whereas Myanmar had the highest cerebrovascular mortality, particularly stroke (20%). Neoplasms (cancers) ranked first as a cause of death in Thailand (21%) and Cambodia (17%). The most common cancers were lung, liver, stomach, and upper respiratory tract. Chronic respiratory diseases (eg, asthma and chronic obstructive pulmonary diseases) were most prevalent in Bangladesh (10%) and least common in Cambodia (1%). Injuries (eg, road traffic accidents, falls, suicides, and drownings) were major contributors to mortality in all countries (7–13% of all deaths), with most of these deaths (73%) occurring in males (appendix 7 p 12). Genito-urinary conditions (eg, nephrotic syndrome and nephritis) were most common in Thailand and Myanmar. Diarrhoeal diseases (eg, typhoid, paratyphoid, and gastroenteritis) were notable in Bangladesh (4%) and Laos (4%). Digestive diseases (eg, alcoholic liver disease and hepatitis) were highest in Myanmar (16%), Cambodia (14%), and Laos (13%). Acute respiratory diseases, such as pneumonia, were more frequent in Laos and Thailand (5% each). Tuberculosis was one of the top ten causes of death in all countries except Thailand. The CSMFs for diabetes ranged from 6% in Laos and Cambodia to 2% in Myanmar and Thailand. Child mortality was mainly from acute respiratory infections, pneumonia, diarrhoeal diseases, and tuberculosis (CSMFs were not calculated due to the small number of cases). There were 13 maternal deaths recorded among the 3413 total deaths (0·4%). Sex-disaggregated CSMFs (appendix 7 p 12) revealed males with higher injury and cardiovascular-related mortality, and females had similar or slightly higher CSMFs for neoplasms, diabetes, and cerebrovascular diseases.

NCDs accounted for most YLLs across all five countries (table 3). Myanmar, which had the highest crude death rate (19 per 1000 population), also recorded the highest YLL rate (30 827 per 100 000), driven by NCDs (56% of YLLs) and injuries (18%). Laos, with the second-highest death rate (nine per 1000), faced a dual burden, where NCDs constituted 46% of YLLs, and communicable, maternal, perinatal, and nutritional conditions accounted for 32%. In Cambodia, NCDs represented 68% of YLLs, whereas injuries accounted for a further 21%. In Thailand, NCDs were responsible for 65% of YLLs and injuries for 16%. Similarly, in Bangladesh, NCDs accounted for 56% of YLLs and 11% for injuries, resulting in a total of 9926 YLLs per 100 000 population. Age and YLL distribution by major cause group is shown in appendix 7 (p 13).

Health-care-seeking behaviour during the deceased’s final illness, highlighting differences in health-care access, types of facilities used, and barriers such as travel time and the perceived financial burden on households have been summarised (table 4). In Cambodia and Laos, over 75% of cases did not seek formal health care. Among health-seeking individuals, Thailand had the highest health-care use with 83% attending government facilities, followed by Myanmar and Laos at 62% each and Cambodia and Bangladesh at only 7% each. Private health care was most used in Cambodia (40%). Of home deaths, 15–26% had hospitalisation, with patients discharged before death. Many in Bangladesh (89%) and Cambodia (52%) relied on alternative providers (eg, traditional healers, religious leaders, pharmacies, friends, and relatives). Travel time to health-care facilities was reported as a barrier to access for 38% of deaths in Cambodia and 23% in Bangladesh, but only 5% in Thailand and 6% in Myanmar. The financial burden of health-care affected 47% of Bangladeshi and 40% of Cambodian families, compared with 17% in Myanmar, but this was less common in Laos (14%) and Thailand (4%).

Initial physician discrepancies ranged from 15% to 30% of all cases. Of these discrepant cases, 85–90% were successfully reconciled while adjudication by a third coder was required in 10% and 15% (appendix 7 p 14).

## Discussion

This study explored mortality patterns across rural areas in five southeast Asian countries, highlighting leading cause of death, health-care access disparities, and influencing factors. Findings reveal that even in rural areas, NCDs, including cardiovascular diseases, cancers, injuries, chronic respiratory conditions, and diabetes, are the leading causes of death, whereas tuberculosis, pneumonia, and diarrhoeal diseases remain significant health challenges. These results align with previous regional studies and the Global Burden of Diseases, Injuries, and Risk Factors Study (GBD) 2021 estimates.^18–20^ A major gap in death-related documentation underscores the need to strengthen civil registration and vital statistics systems, particularly in rural areas. Key actions include raising public awareness, simplifying administrative processes, enforcing legal mandates, and enhancing coordination with health-care providers for death certification.^21,22^

Across the sites, fewer neonatal, child, and maternal deaths were recorded than expected, possibly a reflection of successful maternal and child health interventions implemented before the COVID-19 pandemic and demographic changes such as declining fertility. In Thailand, in particular, this was likely further reinforced by increased health-care investments. WHO has reported a historic low in global under-five mortality in 2022.^23^ However, under-ascertainment of neonatal and infant deaths remains likely in community-based surveillance, especially when deaths occur shortly after birth or outside health facilities. These methodological limitations might thus underestimate mortality in younger age groups. Nevertheless, stark disparities across provinces, countries, and regions^24^ indicate the need for targeted actions to meet the Sustainable Development Goal, for reducing maternal and child mortality.

The study findings might be affected by under-reporting, particularly among women, children, and marginalised groups. In many rural southeast Asian communities, cultural norms that prioritise men as household heads, limited health-care access, and weaker community-based death notification systems might all contribute to such biases.^25^ Although global analyses suggest minimal sex bias in death registration, some countries in south and southeast Asia—including Bangladesh, India, Indonesia, Laos, and Viet Nam—have under-reported female deaths and this might affect data accuracy.^25^ In our dataset, the skewed sex distribution and lower injury mortality among women (15–64 years) suggest possible under-reporting or misclassification. Sensitive causes, such as suicide, HIV/AIDS, or alcohol-related liver disease, might also be under-reported due to stigma or cultural taboos, potentially affecting estimates for communicable, maternal, perinatal, and nutritional conditions.

Health-care access in the study region is affected by the availability of government-run public health facilities and the financial costs of formal health services.^26^ The present study revealed a common reliance on informal sources, such as traditional healing practices, over formal health-care services. However, national health and social insurance schemes in Laos and Thailand appear to reduce such financial burdens.

Our study has several strengths. It covered five culturally and geographically diverse countries in southeast Asia, offering a regional overview of mortality in rural populations. The use of multiple community and administrative data sources for the validation of death report improved completeness. Public engagement before data collection enhanced cultural sensitivity and participation rates. Although about 9% of reported deaths did not have verbal autopsies (due to family migration or refusal), this is unlikely to introduce significant bias. Discrepancies in double-coded cause-of-death assignment were within an acceptable range, indicating robust data collection and standardised physician training. Less than 5% of deaths of people younger than 70 years were classified as unspecified causes, indicating a reasonable quality in the electronic verbal autopsies. Previous research confirms that physician-coded methods outperform algorithmic models.^27^ In our study, this was reinforced by the use of well validated tools, adherence to WHO guidelines, local-language training, and rigorous quality control. Grouping causes into broad categories (eg, cardiovascular diseases rather than ischaemic heart disease) minimised misclassification and improved reliability in identifying major health burdens.

Several important limitations are acknowledged. Conflict-affected villages in Bangladesh (Roowangchhari, Bandarban District) and Myanmar (southern areas of Myawaddy township, Karen State), and all urban areas were excluded due to security concerns and rural study focus. Results are therefore not generalisable to urban or conflict settings. Completeness of death reporting could not be measured due to the absence of reliable death registries. As a result, total mortality and CSMFs might be underestimated. Nearly half (44%) of verbal autopsies were done 6–12 months after death, potentially introducing recall bias particularly for cases involving vague or overlapping symptoms. COVID-19-related deaths were few (<1%; data collection resumed after the COVID-19 pandemic) but were likely underestimated due to limited testing in rural areas, possible misclassification of respiratory-related deaths, stigma, and low health-seeking behaviour. Furthermore, use of broad diagnostic categories, such as cardiovascular disease, reduced precision for disease-specific health planning. Some discrepancies between study-derived CSMFs and WHO or GBD estimates, which apply more granular classifications, suggest the need for contextual interpretation and highlight the importance of verbal autopsy alongside other mortality data sources. Variation in crude death rates (three to 19 per 1000) across the sites reflects differences in health system performance, population structure^28^ and notification completeness, and methodological differences in death identifications, limiting direct cross-country comparisons. Although not nationally representative, the findings still offer important insights into rural mortality and health inequalities often missed by national statistics. The 2-year data collection period limited us from analysing mortality trends but findings are broadly consistent with regional estimates.^18–20,29^

Even without nationwide data, the rural mortality data can guide site-specific strategies. Myanmar and Laos need health-care reforms addressing NCDs, communicable diseases, and road safety. Bangladesh and Cambodia must prepare for rising NCD burdens, whereas Thailand should sustain efforts in reducing NCDs and injuries. Effective strategies include tobacco and alcohol control, cancer screening, vaccinations, healthy lifestyle promotion, improved road safety, health systems strengthening, and regional collaboration.

Males aged 30–59 years had a 60% higher mortality risk than females, due to biological, behavioural, and societal factors.^30^ Sex-disaggregated CSMFs provide evidence that the leading causes of death differ by sex and age, with males experiencing higher mortality from cardiovascular disease, respiratory infections, alcoholic liver diseases, and injuries. These differences highlight the need for sex-specific and age-specific policies, research, and interventions to promote health equity, particularly given the pronounced disparities in injury-related mortality between males and females.

In summary, this study identifies the leading causes of death and examines health-care access, and death documentation practices in rural southeast Asia. Although non-communicable diseases were the leading causes of death across sites, infectious diseases and accidents remain common, underscoring a dual burden that requires context-specific strategies encompassing prevention, early detection, treatment, and accessible health-care delivery. Electronic verbal autopsy was shown to be a feasible and scalable tool for improving mortality data in LMIC settings and could be integrated into routine health surveillance. Strengthening death registration and certification particularly in Myanmar, Bangladesh, and Laos is an important priority. Furthermore, the high proportion of individuals who did not access health care before death emphasises the need to better understand and address barriers to care in these rural populations.

## Supplementary Material

Supplementary info and appendices

## Figures and Tables

**Table 1:** Background characteristics of verbal autopsies

	Bangladesh (n=510)	Myanmar (n=296)	Thailand (n=894)	Laos (n=1492)	Cambodia (n=221)
Catchment population	115 962	21 471	117 363	161 113	68 089
Total number of deaths reported	557	411	955	1584	246
Total number of verbal autopsies	510	296	894	1492	221
Verbal autopsy coverage	91%	72%	93%	94%	89%
Age group distribution, n=3413					
Adolescents and adults (aged >12 years)	476/510 (93%)	285/296 (96%)	887/894 (99%)	1379/1492 (92%)	218/221 (99%)
Children (aged 29 days to 12 years)	27/510 (5%)	8/296 (3%)	6/894 (1%)	101/1492 (7%)	3/221 (1%)
Neonates (aged 0-28 days)	7/510 (1%)	3/296 (1%)	1/894 (<1%)	12/1492 (1%)	0/221
Sex					
Male	300/510 (59%)	181/296 (61%)	536/894 (60%)	902/1492 (60%)	133/221 (60%)
Female	210/510 (41%)	115/296 (39%)	358/894 (40%)	590/1492 (40%)	88/221 (40%)
Place of death					
Home	330/510 (65%)	196/296 (66%)	582/894 (65%)	1293/1492 (87%)	165/221 (75%)
Hospital or health facilities	139/510 (27%)	66/296 (22%)	279/894 (31%)	122/1492 (8%)	41/221 (19%)
Other*	41/510 (8%)	34/296 (11%)	33/894 (4%)	77/1492 (5%)	15/221 (7%)
Smoking, male adults	155/276 (56%)	132/174 (76%)	181/531 (34%)	378/831 (45%)	55/131 (42%)
Alcohol drinking, male adults	20/276 (7%)	121/174 (70%)	235/531 (44%)	280/831 (34%)	40/131 (31%)
Time to interview after death					
<3 months	196/510 (38%)	145/296 (49%)	316/881 (36%)†	430/1488 (29%)†	89/215 (41%)†
3 to <6 months	306/510 (60%)	58/296 (20%)	219/881 (25%)†	406/1488 (27%)†	82/215 (38%)†
6 to 12 months	8/510 (2%)	93/296 (31%)	346/881 (39%)†	652/1488 (44%)†	44/215 (21%)†
Death certificate					
Present	70/510 (14%)	0/296	865/894 (97%)	6/1492 (<1%)	152/221 (69%)
Absent	440/510 (86%)	296/296 (100%)	29/894 (3%)	1468/1492 (98%)	69/221 (31%)
Death registration					
Present	40/510 (8%)	0/296	216/894 (24%)	4/1492 (<1%)	86/221 (39%)
Absent	470/510 (92%)	296/296 (100%)	678/894 (76%)	1488/1492 (>99%)	135/221 (61%)


Data are n, %, or n (%). We presented the proportions of the variable based solely on “yes” responses. Smoking and alcohol use were not reported for female deaths. This omission reflects both cultural norms and low prevalence in rural southeast Asian communities, where such behaviours are socially discouraged or stigmatised among women. National surveys, previous studies (eg, WHO Global Health Observatory data), and this study indicate female smoking rates below 3%, with similarly low and under-reported alcohol consumption. The time interval between the date of death and the date of the verbal autopsy interview was categorised into three groups for analysis: <3 months, 3 to <6 months, and 6–12 months after death. *Accidental places and on the way to health facilities were included. †The time of interview after death could not be determined for 13 cases in Thailand, four cases in Laos, and six cases in Cambodia because the date of death was unavailable (these deaths occurred in recent months, but the exact dates were not recorded); therefore, these cases were excluded from the calculation.

**Table 2:** Leading causes of deaths according to cause-specific mortality fractions, ranked from one to ten

	Bangladesh			Myanmar			Thailand			Laos			Cambodia	
	Causes of deaths	CSMF		Causes of deaths	CSMF		Causes of deaths	CSMF		Causes of deaths	CSMF		Causes of deaths	CSMF
1	Cardiovascular*	15 (12 to 18)		Cerebrovascular*	20 (16 to 24)		Neoplasms*	21 (18 to 23)		Digestive related*	13 (11 to 15)		Neoplasms*	17 (12 to 22)
2	Cerebrovascular*	14 (10 to 17)		Digestive related*	16 (11 to 21)		Accidents and injuries	13 (10 to 15		Cardiovascular*	7 (6 to 9)		Digestive related*	14 (10 to 19)
3	Neoplasms*	14 (11 to 16)		Accidents and injuries	12 (7 to 18)		Cerebrovascular*	9 (7 to 11)		Neoplasms*	7 (5 to 9)		Cardiovascular*	11 (7 to 15)
4	Chronic respiratory*	10 (7 to 13)		Neoplasms*	11 (9 to 14)		Cardiovascular*	9 (7 to 11)		Diabetes*	6 (5 to 8)		Cerebrovascular*	11 (7 to 15)
5	Accidents and Injuries	7 (5 to 9)		Genito-urinary related*	4 (1 to 7)		Genito-urinary related*	8 (6 to 10)		Accidents and injuries	6 (5 to 8)		Accidents and injuries	10 (6 to 14)
6	Genito-urinary related*	5 (4 to 7)		Chronic respiratory diseases*	3 (1 to 5)		Digestive related*	8 (6 to 9)		Genito-urinary*	6 (4 to 7)		Diabetes*	6 (3 to 9)
7	Diarrhoea related†	4 (2 to 6)		Tuberculosis†	3 (–0·2 to 6)		Chronic respiratory diseases*	6 (4 to 8)		Pneumonia†	5 (4 to 6)		Genito-urinary*	5 (2 to 9)
8	Digestive related*	3 (2 to 5)		Cardiovascular*	2 (0·7 to 4)		Pneumonia†	5 (3 to 7)		Tuberculosis†	5 (4 to 6)		Tuberculosis†	4 (1 to 7)
9	Pneumonia†	3 (1 to 5)		Diabetes*	2 (0·5 to 3)		Diabetes*	2 (1 to 3)		Cerebrovascular*	4 (3 to 5)		Pneumonia†	3 (0·3 to 5)
10	Tuberculosis†	2 (1 to 4)		Diarrhoea related†	1 (0·1 to 2)		Diarrhoea related†	2 (1 to 3)		Diarrhoea related†	4 (3 to 5)		Chronic respiratory diseases*	1 (0·1 to 3)

Data are % (95% CI). Unknown causes (excluding senility) as percentage of total deaths within broader category of undetermined or ill-defined deaths (not ranked): three for Bangladesh, three for Myanmar, one for Thailand, five for Laos, and one for Cambodia. CSMF=cause-specific mortality fractions.

* Non-communicable diseases.

† Communicable, maternal, perinatal, and nutritional causes.

**Table 3:** Years of life lost across the studies, per 100 000 population, by cause of death groups

	Bangladesh (n=9926)	Myanmar (n=30 827)	Thailand (n=14 412)	Laos (n=24 416)	Cambodia (n=6435)
Non-communicable diseases	5569 (56%); 55–57	17 231 (56%); 55–56	9427 (65%); 64–66	11 171 (46%); 45–46	4394 (68%); 67–69
Communicable, maternal, perinatal, and nutritional causes	2813 (28%); 27–29	5841 (19%); 18–19	2246 (16%); 15–16	7995 (33%); 32–33	528 (8%); 7–9
Accidents and injuries	1113 (11%); 10–12	5660 (18%); 17–19	2372 (16%); 15–17	2634 (11%); 10–11	1337 (21%); 19–21
Undetermined or ill-defined conditions	431 (4%) 3–5	2095 (7%); 6–9	367 (3%); 2–3	2616 (11%); 10–11	176 (3%); 2–3

Data are n (%); 95% CI.

**Table 4:** Frequency of health-care access as reported by verbal autopsy

	Bangladesh	Myanmar	Thailand	Laos	Cambodia
Health care sought during final illness*	136/510 (27%)	132/296 (45%)	283/894 (32%)	333/1492 (22%)	55/221 (25%)
Types of health-care facilities used					
Only government hospitals or health centres	10/136 (7%)	82/132 (62%)	236/283 (83%)	205/333 (62%)	4/55 (7%)
Only private hospitals or physicians Informal sources	5/136 (4%)	22/132 (17%)	3/283 (1%)	14/333 (4%)	22/55 (40%)
Traditional healers and religious leaders	2/136 (1%)	24/132 (18%)	37/283 (13%)	108/333 (32%)	22/55 (40%)
Friends and relatives	28/136 (21%)	17/132 (13%)	33/283 (12%)	0/333	2/55 (4%)
Pharmacy	28/136 (21%)	2/132 (2%)	0/283	22/333 (7%)	5/55 (9%)
Others†	121/136 (89%)	28/132 (21%)	44/283 (16%)	114/333 (34%)	29/55 (53%)
Travel to health facilities	252/510 (49%)	66/296 (22%)	493/894 (55%)	652/1492 (44%)	73/221 (33%)
Travel time >2 h to health-care facilities	117/510 (23%)	18/296 (6%)	47/894 (5%)	175/1492 (12%)	86/221 (39%)
Burden of health-care cost on their households	242/510 (47%)	53/296 (18%)	44/894 (5%)	213/1492 (14%)	90/221 (41%)

Data are n/N (%). We presented the proportions of the variable based solely on “yes” responses. Column totals might exceed 100% due to multiple responses permitted for types of health-care facilities used.

* Refers to the health care sought during the final episode of illness, regardless of whether any previous health care had been received.

† Includes a combination of government or private facilities, along with informal sources such as religious leaders, traditional healers, pharmacies, relatives, friends, and similar sources.

## Data Availability

Data collected for the study, including de-identified individual participant data, will be made available to others. These data will be available with publication via the MORU data sharing committee (datasharing@tropmedres.ac). Access will be provided for analysis by bona fide researchers with or without investigator support, after approval of a proposal, and upon a signed data access agreement.

## Research in context

### Evidence before this study

Mortality estimates in southeast Asia have largely relied on modelled data produced by the Global Burden of Disease, Injuries, and Risk Factors Study (GBD), WHO, and the UN. According to GBD 2021, the leading causes of death in the region include stroke, ischaemic heart disease, chronic respiratory diseases, and cancers, highlighting the rising toll of non-communicable diseases (NCDs). However, these estimates are derived from statistical models that often rely on incomplete or outdated data, particularly in low-income and middle-income country settings. We conducted a PubMed search for articles published before March 1, 2025, using the terms “verbal autopsy”, “cause of death”, and “mortality”, in combination with country-specific terms: “Bangladesh”, “Myanmar”, “Lao”, “Thailand”, and “Cambodia”. Of the seven articles found, the majority of studies emphasised the dominance of NCDs, poor death certification coverage, and the feasibility of verbal autopsy tools, either physician-coded or automated, for identifying probable causes of death in rural populations. WHO reports that less than 25% of deaths in the region were documented in civil registration systems in 2020, with even fewer having medically certified causes. The data gap is starkest in rural areas, where more than 70% of deaths occur outside health facilities, leaving them uncounted in official statistics. Studies from 2020 to 2024 highlight that less than 10% of rural deaths in parts of southeast Asia are medically certified, making it necessary to rely on extrapolated models that might not reflect local realities. Without granular, community-level data, interventions risk being misaligned with the true disease burden.

### Added value of this study

This study is the first to implement an electronic verbal autopsy across five southeast Asian countries, documenting mortality patterns in rural communities not covered by existing surveillance networks. Our findings highlight substantial gaps in death registration, emphasising the need for strengthened reporting systems. The study also reveals that non-communicable diseases dominate mortality in these rural settings, and that health-care-seeking patterns vary widely across countries. These differences offer important insights into how health system structure, accessibility, affordability, and cultural factors shape health-care use, providing evidence to inform targeted health system interventions and policy planning. Although our study does not examine mortality trends over time, the findings highlight the substantial current burden of NCDs in rural southeast Asia, consistent with GBD regional estimates and recent verbal autopsy studies.

### Implications of all the available evidence

Rising NCDs in rural areas indicate a call for policies that integrate chronic disease management with infectious disease control, tailored to local health-care access patterns. Our study shows that electronic verbal autopsy tools can bridge mortality data gaps in resource-limited settings. Governments should strengthen civil registration systems and redirect resources towards rural NCD prevention and primary care.
