# Supplementary material for: Causes of death in rural southeast Asia by electronic verbal autopsy: a population-based observational study
Source: Lancet Glob Health. Author manuscript; Available in PMC 2026 Oct 30. (PMC7619658; doi:10.1016/S2214-109X(25)00362-6)
Supplement: Supplementary file 1 [file EMS219658-supplement-Supplementary_info_and_appendices.pdf]

နောက်ခံအကြောင်းအရာ

ဝင်ငွေ နည်းပါးသော နှင့် ဝင်ငွေ အလယ်အလတ်ရှိသော အရှေ့တောင်အာရှနိုင်ငံများတွင် သေဆုံးမှုအများစုသည် ကျန်းမာရေးစောင့်ရှောက်မှုစနစ်၏ ပြင်ပ၌ ဖြစ်ပွားလေ့ရှိပြီး ဆေးပညာအရ တရားဝင် အသိအမှတ်ပြုထားသော သေဆုံးရသည့်အကြောင်းရင်း မရှိကြပါ။ ကျွန်ုပ်တို့သည် ဒေသတွင်း ကျေးလက်ဒေသများတွင် သေဆုံးရသည့် အရင်းခံ အကြောင်းတရားများကို ဖော်ထုတ်ရန်နှင့် ဆုံးရှုံးခဲ့ရသော ဘဝသက်တမ်းနှစ်များကို အသုံးပြု၍ အရွယ်မတိုင်မီ သေဆုံးနှုန်းကို ခန့်မှန်းနိုင်ရန် နှုတ်ဖြေဖြင့် သေဆုံးမှုအကြောင်းရင်း စစ်တမ်းကောက်ယူခြင်းဆိုင်ရာ သုတေသနလေ့လာမှုတစ်ခုကို ပြုလုပ်ခဲ့ပါသည်။

သုတေသနပြုသည့် နည်းစနစ်များ

၂၀၂၁ ခုနှစ်မှ ၂၀၂၄ ခုနှစ်အတွင်း ဘင်္ဂလားဒေ့ရှ်၊ ကမ္ဘောဒီးယား၊ လာအိုပြည်သူ့ဒီမိုကရက်တစ်သမ္မတနိုင်ငံ၊ မြန်မာနှင့် ထိုင်းနိုင်ငံတို့ရှိ ကျေးရွာပေါင်း ၅၁၀ ရွာတွင် သတင်းရရှိသော သေဆုံးမှုများအတွက် အီလက်ထရွန်နစ်စနစ်ကိုအသုံးပြု၍ နှုတ်ဖြေဖြင့် သေဆုံးမှုအကြောင်းရင်း စစ်တမ်းများကို မေးမြန်းကောက်ယူခဲ့ပါသည်။ ကမ္ဘာ့ကျန်းမာရေးအဖွဲ့၏ ၂၀၁၆ ခုနှစ် နှုတ်ဖြေဖြင့် သေဆုံးမှုအကြောင်းရင်း စစ်တမ်းမေးခွန်းလွှာကို အသုံးပြု၍ လေ့ကျင့်သင်ကြားထားသည့် ကွင်းဆင်းဝန်ထမ်းများမှ တွေ့ဆုံမေးမြန်းမှုများ ပြုလုပ်ခဲ့ပြီး၊ ဒေသခံဆရာဝန်များမှ သေဆုံးရသည့်အကြောင်းရင်းများကို အိုင်စီဒီ-၁၀ (ICD-10) ကုဒ်နံပါတ်များဖြင့် သီးသန့်လွတ်လပ်စွာ သတ်မှတ်ပေးခဲ့ကြပါသည်။

သုတေသနတွေ့ရှိချက်များ

သတင်းရရှိသော သေဆုံးမှုအားလုံး၏ ၉၀% ကျော်အတွက် နှုတ်ဖြေဖြင့် သေဆုံးမှုအကြောင်းရင်း စစ်တမ်းကောက်ယူနိုင်ခဲ့ပါသည်။ စစ်တမ်းကောက်ယူခဲ့သည့် သေဆုံးသူ ၃၄၁၃ ဦးအနက် ၆၀% မှာ အမျိုးသားများဖြစ်ကြပြီး ၉၅% မှာ အသက် ၁၂ နှစ်အထက် ဖြစ်ကြပါသည်။ သုတေသနပြုလုပ်သည့် နေရာများအားလုံးတွင် သေဆုံးမှုများ၏ ၆၄-၈၆% သည် နေအိမ်၌ ဖြစ်ပွားခဲ့ခြင်း ဖြစ်ပါသည်။ မကူးစက်နိုင်သော ရောဂါများသည် အဓိကသေဆုံးရသည့် အကြောင်းရင်းများဖြစ်ပြီး သုတေသနပြုလုပ်သည့် နေရာများအလိုက် လာအိုနိုင်ငံတွင် ၅၄% မှ ကမ္ဘောဒီးယားနိုင်ငံတွင် ၇၀% အထိ ရှိပြီး ၎င်းတို့အနက် နှလုံးသွေးကြောဆိုင်ရာရောဂါ၊ ဦးနှောက်သွေးကြောဆိုင်ရာရောဂါ၊ ကင်ဆာရောဂါနှင့် အစာခြေစနစ်ဆိုင်ရာ ရောဂါများမှာ အဖြစ်အများဆုံးဖြစ်သည်။ မိခင်၊ အာဟာရနှင့်ဆိုင်သောရောဂါများနှင့် ကူးစက်ရောဂါများ၊ အဓိကအားဖြင့် အသက်ရှူလမ်းကြောင်းဆိုင်ရာ ကူးစက်ရောဂါများ၊ ဝမ်းလျှောရောဂါနှင့် တီဘီရောဂါတို့သည် သုတေသနပြုလုပ်သည့် နေရာအသီးသီးရှိ သေဆုံးမှု စုစုပေါင်း၏ ၁၁-၂၆% အထိ ရှိပါသည်။ မတော်တဆထိခိုက်မှုများနှင့် ဒဏ်ရာရရှိမှုများကြောင့် သေဆုံးခြင်းသည် ၇-၁၃% အထိ ရှိခဲ့ပြီး အဓိက သေဆုံးရသည့် အကြောင်းရင်း ထိပ်ဆုံး ၅ ခုတွင် ပါဝင်ခဲ့ပါသည်။ သုတေသနပြုလုပ်ခဲ့သော နိုင်ငံအားလုံးတွင် သေဆုံးမှုဆိုင်ရာ မှတ်တမ်းများ ပြုစုရာ၌

အဓိကလိုအပ်ချက်များ ဆက်လက်ရှိနေဆဲဖြစ်ပြီး၊ ရောဂါအလိုက် သေဆုံးမှု အကြောင်းရင်းများမှာလည်း သုတေသနပြုလုပ်ခဲ့သော နေရာကွာခြားမှုအလိုက် ကွဲပြားလျက်ရှိပါသည်။

ကောက်နှုတ်သုံးသပ်ချက်

နိုင်ငံအများအပြားတွင် ပူးပေါင်းပြုလုပ်ခဲ့သော ဤသုတေသနလေ့လာမှုသည် အရှေ့တောင်အာရှရှိ ကျေးလက်ဒေသများတွင် မကူးစက်နိုင်သော ရောဂါများ၏ ဝန်ထုပ်ဝန်ပိုးမှာ သိသာစွာ ကြီးမားနေဆဲဖြစ်သကဲ့သို့၊ ကူးစက်နိုင်သော ရောဂါများမှာလည်း ဆက်လက်တည်ရှိနေဆဲဖြစ်ကြောင်းကို မီးမောင်းထိုးပြလျက်ရှိပါသည်။ ထို့ကြောင့် ဤရောဂါအခြေအနေ နှစ်မျိုး၏ ဝန်ထုပ်ဝန်ပိုးကို လျှော့ချနိုင်ရန်အတွက် ပိုမိုကောင်းမွန်တိကျသော သေဆုံးမှုဆိုင်ရာ အချက်အလက်များနှင့် ပိုမိုကောင်းမွန်သော ကျန်းမာရေးစောင့်ရှောက်မှု ရရှိရေး လိုအပ်နေကြောင်းကိုလည်း ဤသုတေသနလေ့လာမှုမှ ဖော်ပြနေပါသည်။

တၢ်ဃိထံၣ်သ့ၣ်ညါအဂ့ၢ်အကျိၤလၢညါ

ကမျၢၢ်အိၣ်လၢထံကီၢ်လၢကျိၣ်စ့တၢ်ဟဲနုာ်စုၤမ့တမ့ၢ်အိၣ်ဖဲအကြၢးလၢကီၢ်အ့ရှၢၢ်မုၢ်ထီၣ်ကလံၤထံးသ့ၣ်တဖၣ် အံၤ အါဒၣ်တၢ်တက့ၢ်တမ့ၢ်သံလၢတၢ်ဆါဟံၣ်အပူၤဖဲအထီၣ်တၢ်ဆါဟံၣ်အခါဘၣ်အဃိကသံၣ်သရၣ်လံာ်အုၣ်ကီၤသး လၢတၢ်သံတၢ်ပှၢ်အဂ့ၢ်အတၢ်မၤနီၣ်တအိၣ်ဘၣ်.

ပမၤဝဲတၢ်ဃိထံသံကွၢ်သမံသမိးလၢထးခိၣ်ဘၣ်ဃးတၢ်သံအဂ့ၢ် (verbal autopsy) လၢခီခိၣ်တၢ်လီၢ်ဒ်သိးကဃု သ့ၣ်ညါဘၣ်ဃးတၢ်ဆူးတၢ်ဆါပနီၣ်အဂ့ၢ်အိၣ်မနုၤသ့ၣ်တဖၣ်လၢပှၤသံလဲၣ် ဒီးဒ်သိးကထိၣ်တယာ်ကွၢ်က့ၤသး သမူအတၢ်လဲၤအနံၣ်အလါဒီးတၢ်သံဆိအဂ့ၢ်အကျိၤ (Years of Life Lost) န့ၣ်လီၤ.

တၢ်ဃိထံအကျိၤအကျဲအသနူ

စးထီၣ်လၢ ၂၀၂၁ တုၤလၢ ၂၀၂၄ နံၣ်အတီၢ်ပူၤ ပဆိးကါဝဲအံးလဲးထြိနံးတၢ်ဃိထံတၢ်သံကွၢ်သမံသမိးလၢထးခိၣ် ဘၣ်ဃးဒီးပှၤသံအဂ့ၢ်လၢသဝီ ၅၁၀ ဖျၢၣ်လၢအပၣ်ဃုာ် ကီၢ်ဘ့ကလါဒ့း,ကီၢ်ခဲၤဘီဒံယါ, ကီၢ်လါအိၣ်, ကီၢ်ပယီၤ ဒီး ကီၢ်ကိုၣ်တဲၣ်န့ၣ်လီၤ. တၢ်ဆိးကါဝဲဒၣ်တၢ်ပာ်ပနီၣ်တၢ်သံကွၢ်လၢအမ့ၢ် ၂၀၁၆ ဟီၣ်ခိၣ်ဒီဖျၢၣ်ဆူၣ်ချ့ကရၢအတၢ်သံ ကွၢ်သမံသိးလၢထးခိၣ်ဘၣ်ဃးတၢ်သံအဂ့ၢ် ဆိးကါဝဲဒၣ်ပျိပူၤပှၤမၤတၢ်ဖိလၢအဒိးန့ၢ်ဘၣ်တၢ်မၤလိမူးမူးနီၢ်နီၢ်ဒ်သိး ကသံကွၢ်ပှၤလၢအဟံၣ်ဖိဃီဖိဆူးကွံာ်တ့ၢ်လံအသးတဖၣ်ဘၣ်ဃးဒီးတၢ်ဆါပနီၣ်အဂ့ၢ်အကျိၤဒီးတၢ်ဆိးကါ စ့ၢ်ကီးလီၢ်ကဝီၤကသံၣ်သရၣ်လၢကကွၢ်ထံကဒါက့ၤတၢ်ဆါအတၢ်ဂ့ၢ်အကျိၤဒ်ကပာ်ဖျါထီၣ်တၢ်ထံၣ်လၢဘၣ်ထွဲ ဒီးတၢ်ဆါပနီၣ်လၢအဒုးအိၣ်ထီၣ်တၢ်သံလၢဆိးကါဝဲဟီၣ်ခိၣ်ဖျၢၣ်သနူအကျိၤအကျဲလၢတၢ်နီၤဖးတၢ်ဆါအကလုာ် သနိ (ICD-10 codes) န့ၣ်လီၤ.

တၢ်အစၢတဖၣ်

လၢတၢ်ပာ်ဖျါပှၤသံနီၣ်ဂံၢ်အဖီခိၣ် အါန့ၢ်ဒံး ၉၀ အမျၢ်ကယၤ အံၤဘၣ်တၢ်သံကွၢ်သမံသိးလၢထးခိၣ်ဘၣ်ဃး တၢ်သံအဂ့ၢ်န့ၣ်လီၤ. လၢပှၤသံသးနံၣ်အိၣ် ၁၂ နံၣ်ဆူအဖီခိၣ်အနီၣ်ဂံၢ် ၃,၄၁၃ အကျါ ပိာ်ခွါအိၣ်အမျၢ်ကယၤ ၆၀ ဒီးပိာ်မုၣ်အိၣ်အမျၢ်ကယၤ ၉၅ န့ၣ်လီၤ. လၢတၢ်ဃိထံတၢ်လီၢ်ထံကီၢ်ကိးဘ့ၣ်ဒဲးအကျါ ၆၄-၈၆ မျၢ်ကယၤပာ်ဖျါ ဝဲလၢသံလၢဟံၣ်လီၤ. တၢ်သံတၢ်ပှၢ်အါကတၢၢ်အဂ့ၢ်န့ၣ်မ့ၢ်ဝဲခီဖျိတၢ်ဆါလၢအဘၣ်ကူဘၣ်ဂာ်တၢ်တသ့ဘၣ် အိၣ်ဝဲ ၅၄ မျၤကယၤလၢ ကီၣ်လါအိၣ်, တုၤလၢ ၇၀ မျၤကယၤ လၢကီၢ်ခဲၤဘီဒံယါန့ၣ်လီၤ. တၢ်ဆါသ့ၣ်တဖၣ်မ့ၢ်ဝဲ သးဖျၢၣ်(လီၤ)သွံၣ်ကျိၤတၢ်ဆါ, ခိၣ်နူးသွံၣ်ကျိၤတၢ်ဆါ, ခဲစၢၢ်တၢ်ဆါ, ဒီး တၢ်အီၣ်ကျိၤတၢ်ဆါအအါကတၢၢ်န့ၣ် လီၤ.

တၢ်ဃိထံအစၢအခီပညီ

တၢ်ဃိထံသ့ၣ်ညါလၢတၢ်မၤအီၤလၢကီၢ်တဘျုးဘ့ၣ်အံၤဒုးနဲၣ်ဖျါထီၣ်ဝဲလၢအ့ရှၢၢ်မုၢ်ထီၣ်ကလံၤထံးခီခိၣ်တၢ်လီၢ် အံၤတၢ်ဆါလၢအဘၣ်ကူဘၣ်ဂာ်တၢ်တသ့အံၤမ့ၢ်တၢ်ကီတၢ်ခဲဖးဒိၣ်လၢအကဲထီၣ်အသးအါထဲသိးသိးဒီးတၢ်ဆါ လၢအဘၣ်ကူဘၣ်ဂာ်တၢ်သ့သ့ၣ်တဖၣ်န့ၣ်လီၤ.

တၢ်ပာ်ဖျါအံၤအရှ့ဒိၣ်လၢတၢ်ကမၤဂ့ၤထီၣ်တၢ်မၤနီၣ်ဘၣ်ဃးတၢ်သံတၢ်ပှၢ်အဂ့ၢ်အကျိၤဒီးမၤဂ့ၤထီၣ်ဆူၣ်ချ့ တၢ်အံးထွဲကွၢ်ထွဲအကျိၤအကျဲဒ်သိးကမၤလီၤစှၤတၢ်ဆါခံကလုာ်လၢာ်အံၤန့ၣ်လီၤ.

পটভূমি দক্ষিণ- পূর্ব এশিয়ার নিম্ন এবং মধ্যম আয়ের দেশগুলোতে বেশিরভাগ মৃত্যু স্বাস্থ্যসেবা ব্যবস্থার বাইরে ঘটে থাকে যেখানে মৃত্যুর কারণ সংক্রান্ত মেডিক্যাল সার্টিফিকেট থাকে না। আমরা এই অঞ্চলের গ্রামীণ এলাকায় মৃত্যুর অন্তর্নিহিত কারণগুলি নির্ণয় করতে এবং ইয়ার্স অফ লাইফ লস্ট ব্যবহার করে অকাল মৃত্যুহার অনুমান করার জন্য একটি মৌখিক ময়নাতদন্ত গবেষণা পরিচালনা করেছি।

পদ্ধতি ২০২১ থেকে ২০২৪ সাল পর্যন্ত আমরা বাংলাদেশ, কম্বোডিয়া, লাও পিপল'স ডেমোক্রেটিক রিপাবলিক, মায়ানমার এবং থাইল্যান্ড এর ৫১০ টি গ্রাম এ রিপোর্টকৃত মৃত্যুর ইলেকট্রনিক মৌখিক ময়নাতদন্ত জরিপ পরিচালনা করেছি। বিশ্ব স্বাস্থ্য সংস্থা ২০১৬ মৌখিক ময়নাতদন্ত প্রশ্নাবলী ব্যবহার করে, প্রশিক্ষিত মাঠকর্মীরা সাক্ষাৎকার পরিচালনা করেছেন এবং স্থানীয় চিকিৎসকগণ স্বতন্ত্রভাবে ICD-10 কোড ব্যবহার করে মৃত্যুর কারণ নির্ধারণ করেছেন।

ফলাফল রিপোর্টকৃত মৃত্যুর ৯০% এরও বেশি মৌখিক ময়নাতদন্ত করা হয়েছে। ৩, ৪১৩ জন মৃত্যুর মধ্যে, ৬০% ছিল পুরুষ, এবং ৯৫% ছিলেন ১২ বছরের অধিক বয়সী। বিভিন্ন স্থানে, ৬৪-৮৬% মৃত্যুই বাড়িতে হয়েছিল। মৃত্যুর প্রধান কারণ ছিল অসংক্রামক রোগ, লাওস এ ৫৪% থেকে শুরু করে কম্বোডিয়া তে ৭০%, যার মধ্যে হৃদরোগ,মস্তিষ্কে প্রদাহজনিত রোগ, ক্যান্সার, এবং হজমের সমস্যা সবচেয়ে বেশি পরিলক্ষিত হয়। বিভিন্ন স্থানে, ১১-২৬% মৃত্যুর কারণ ছিল মাতৃত্বজনিত, পুষ্টিগত, এবং সংক্রামক রোগ, প্রধানত শ্বাসতন্ত্রের সংক্রমণ, ডায়রিয়া এবং যক্ষ্মা। দুর্ঘটনা এবং আঘাত মিলে ছিল ৭-১৩% এবং তা শীর্ষ পাঁচটি কারণের মধ্যে স্থান পেয়েছে। মৃত্যুর নথিপত্রের ক্ষেত্রে ব্যপক শূন্যতা সব দেশেই বিরাজমান, যদিও রোগজনিত মৃত্যুর কারণগুলি বিভিন্ন স্থানে বিভিন্নরকম দেখা গেছে।

ব্যাখ্যা এই বহুদেশীয় গবেষণায় দক্ষিণ-পূর্ব এশিয়ার গ্রামীণ এলাকায় বিরাজমান সংক্রামক রোগের পাশাপাশি অসংক্রামক রোগের যে উল্লেখযোগ্য বোঝা রয়েছে তা তুলে ধরা হয়েছে, এই দ্বৈত বোঝা কমাতে মৃত্যুর তথ্য সঠিকভাবে রাখা এবং আরও উন্নত স্বাস্থ্যসেবা পাওয়ার প্রয়োজনীয়তার উপর আলোকপাত করা হয়েছে।

ความเป็นมา ในประเทศเอเชียตะวันออกเฉียงใต้ที่มีระดับรายได้ต่ำและปานกลางการเสียชีวิตส่วนใหญ่เกิดขึ้นนอกระบบบริการสุขภาพ โดยปราศจากการรับรองสาเหตุการเสียชีวิตทางการแพทย์เราได้ดำเนินการศึกษาการสอบสวนการเสียชีวิตโดยการสัมภาษณ์ เพื่อหาสาเหตุการเสียชีวิตในพื้นที่ชนบทของภูมิภาค และเพื่อประเมินอัตราการเสียชีวิตด้วยจำนวนปีที่สูญเสียไปจากการตายก่อนวัยอันควร(YLL)

วิธีการศึกษา ตั้งแต่ปี พ.ศ. 2564 ถึง พ.ศ. 2567 เราได้ดำเนินการสำรวจการสอบสวนการเสียชีวิตโดยการสัมภาษณ์ทางอิเล็กทรอนิกส์สำหรับผู้เสียชีวิตที่ถูกรายงานใน 510 หมู่บ้านในประเทศบังกลาเทศ กัมพูชา สาธารณรัฐประชาธิปไตยประชาชนลาว เมียนมา และประเทศไทย โดยใช้แบบสอบถามการสอบสวนการเสียชีวิตโดยการสัมภาษณ์ขององค์การอนามัยโลก ปี พ.ศ. 2559 เจ้าหน้าที่ภาคสนามที่ผ่านการฝึกอบรมเป็นผู้ดำเนินการสัมภาษณ์ และแพทย์ในพื้นที่เป็นผู้วินิจฉัยและระบุสาเหตุการเสียชีวิตตามดุลยพินิจโดยใช้รหัสโรคสากลฉบับที่ 10 (ICD-10)

ผลการวิจัย มากกว่าร้อยละ 90 ของผู้เสียชีวิตที่ถูกรายงานได้รับการสอบสวนการเสียชีวิตโดยการสัมภาษณ์ ในจำนวนผู้เสียชีวิตทั้งหมด 3,413 ราย พบว่าร้อยละ 60 เป็นเพศชาย และร้อยละ 95 เป็นผู้ที่มีอายุมากกว่า 12 ปี เมื่อพิจารณาจากทุกพื้นที่ศึกษา พบว่าร้อยละ 64-86 ของการเสียชีวิตเกิดขึ้นที่บ้าน กลุ่มโรคไม่ติดต่อเรื้อรังเป็นสาเหตุหลักของการเสียชีวิต อยู่ในช่วง ร้อยละ 54 ในสาธารณรัฐประชาธิปไตยประชาชนลาว ถึงร้อยละ 70 ในประเทศกัมพูชา โดยโรคที่พบบ่อยที่สุด ได้แก่ โรคหัวใจและหลอดเลือด โรคหลอดเลือดสมอง โรคมะเร็ง และโรคเกี่ยวกับระบบทางเดินอาหาร ขณะที่โรคที่เกี่ยวข้องกับการตั้งครรภ์ ภาวะโภชนาการ และโรคติดต่อ โดยเฉพาะโรคติดเชื้อทางเดินหายใจ โรคท้องร่วง และวัณโรค มีสัดส่วนการเสียชีวิตร้อยละ 11-26 ในทุกพื้นที่ศึกษา อุบัติเหตุและการบาดเจ็บคิดเป็นร้อยละ 7-13 และจัดอยู่ในห้าสาเหตุการเสียชีวิตที่พบบ่อยที่สุด ทั้งนี้ยังคงพบช่องว่างสำคัญในการบันทึกข้อมูลการเสียชีวิตในทุกประเทศ และสาเหตุการเสียชีวิตจำเพาะโรคแตกต่างกันไปตามพื้นที่ศึกษา

การตีความผลการวิจัย

การศึกษาในหลายประเทศครั้งนี้แสดงให้เห็นถึงภาระที่โรคไม่ติดต่อเรื้อรังมีความสำคัญในพื้นที่ชนบทของเอเชียตะวันออกเฉียงใต้ ควบคู่ไปกับการดำรงอยู่ของโรคติดต่อเรื้อรัง ซึ่งสะท้อนถึงความจำเป็นในการพัฒนาข้อมูลการเสียชีวิตให้ดีขึ้น และการเข้าถึงบริการด้านสาธารณสุขที่มีประสิทธิภาพมากขึ้น เพื่อลดภาระคู่ของโรคทั้งสองกลุ่มนี้

ບົດຄັດຫຍໍ້

ຄວາມເປັນມາ ປະເທດໃນພາກພື້ນອາຊີຕອນໃຕ້ທີ່ມີລາຍໄດ້ຕໍ່າແລະປານກາງ, ການເສຍຊີວິດສ່ວນໃຫຍ່ເກີດຂື້ນນອກລະບົບສາທາລະນະສຸກ ໂດຍບໍ່ມີການຮັບຮອງຢືນຢັນທາງການແພດເຖິງສາເຫດຂອງການເສຍຊີວິດ. ພວກເຮົາໄດ້ດໍາເນີນການສຶກສາດ້ວຍວິທີການ “ການຫາສາເຫດການຕາຍໂດຍການສອບຖາມ-verbal autopsy” ເພື່ອກໍານົດສາເຫດພື້ນຖານຂອງການເສຍຊີວິດໃນພື້ນທີ່ຊົນນະບົດຂອງພາກພື້ນ ແລະເພື່ອປະເມີນການເສຍຊີວິດກ່ອນໄວອັນຄວນ ໂດຍໃຊ້ຕົວຊີ້ວັດ “ປີຂອງຊີວິດທີ່ສູນເສຍ” (Years of Life Lost - YLL).

ວິທີວິທະຍາ ຈາກປີ 2021 ຫາ 2024 ພວກເຮົາໄດ້ດໍາເນີນການສໍາຫຼວດ “verbal autopsy” ແບບອີເລັກໂທຣນິກສໍາລັບການເສຍຊີວິດທີ່ຖືກລາຍງານໃນ 510 ບ້ານ ໃນປະເທດບັງກະລາເດດ, ກໍາປູເຈຍ, ສາທາລະນະລັດປະຊາທິປະໄຕປະຊາຊົນລາວ, ມຽນມາ ແລະ ປະເທດໄທ. ໂດຍໃຊ້ແບບສອບຖາມ “verbal autopsy” ຂອງອົງການອະນາໄມໂລກ (WHO) ປີ 2016, ພະນັກງານລົງພື້ນທີ່ທີ່ໄດ້ຮັບການຝຶກອົບຮົມໄດ້ດໍາເນີນການສໍາພາດ ແລະທ່ານໝໍທ້ອງຖິ່ນໄດ້ກໍານົດສາເຫດການເສຍຊີວິດຢ່າງເປັນອິດສະຫຼະ ໂດຍໃຊ້ລະຫັດ ICD-10.

ຜົນການສຶກສາ ເກີນ 90% ຂອງການເສຍຊີວິດທີ່ຖືກລາຍງານແມ່ນໄດ້ຖືກດໍາເນີນການສອບຖາມ “verbal autopsy”. ໃນຈໍານວນ 3,413 ການເສຍຊີວິດ, 60% ເປັນເພດຊາຍ ແລະ 95% ມີອາຍຸເກີນ 12 ປີ. ທຸກສະຖານທີ່ສຶກສາ, 64-86% ຂອງການເສຍຊີວິດເກີດຂື້ນຢູ່ບ້ານ. ພະຍາດບໍ່ຕິດຕໍ່ແມ່ນສາເຫດຫຼັກຂອງການເສຍຊີວິດ, ມີສັດສ່ວນຕັ້ງແຕ່ 54% ໃນລາວ ຫາ 70% ໃນກໍາປູເຈຍ, ໂດຍມີພະຍາດຫົວໃຈແລະເສັ້ນເລືອດ, ພະຍາດເສັ້ນເລືອດສະໝອງ, ມະເຮັງ ແລະ ພະຍາດລະບົບລະລາຍອາຫານເປັນພົບຫຼາຍທີ່ສຸດ. ພະຍາດໃນແມ່, ພະຍາດຂາດສານອາຫານ ແລະ ພະຍາດຕິດຕໍ່, ສ່ວນໃຫຍ່ແມ່ນການຕິດເຊື້ອທາງເດີນຫາຍໃຈ, ຖອກທ້ອງ ແລະ ໂຣກວັນນະໂລກ, ຄິດເປັນ 11–26% ຂອງການເສຍຊີວິດທົ່ວທຸກພື້ນທີ່. ອຸປະຕິເຫດ ແລະ ການບາດເຈັບຄິດເປັນ 7-13% ແລະຢູ່ໃນອັນດັບ 5 ສາເຫດຫຼັກ. ການຂາດຂໍ້ມູນການບັນທຶກການເສຍຊີວິດຍັງມີຢູ່ໃນທຸກປະເທດ, ໃນຂະນະທີ່ສາເຫດຈໍາເພາະຂອງການເສຍຊີວິດແຕກຕ່າງກັນໄປຕາມແຕ່ລະພື້ນທີ່.

ການແປຜົນ ການສຶກສາຢູ່ຫຼາຍປະເທດນີ້ໄດ້ສະທ້ອນໃຫ້ເຫັນພາລະສໍາຄັນຂອງພະຍາດບໍ່ຕິດຕໍ່ໃນພື້ນທີ່ຊົນນະບົດຂອງອາຊີຕອນໃຕ້ ຄຽງຄູ່ກັນກັບພະຍາດຕິດຕໍ່ທີ່ຍັງມີຢູ່ ເຊິ່ງສະແດງໃຫ້ເຫັນຄວາມຈໍາເປັນໃນການມີຂໍ້ມູນການເສຍຊີວິດທີ່ດີກວ່າ ແລະ ການເຂົ້າເຖິງການຮັກສາສາທາລະນະສຸກເພື່ອຫຼຸດພາລະຂອງສອງພະຍາດ.

ប្រវត្តិសាវតាប្រទេសដែលមានប្រាក់ចំនូលទាប និងមធ្យមគឺនៅក្នុងតំបន់អាស៊ីអាគ្នេយ៍,ការមរណៈភាពភាគច្រើនគឺកើតឡើងនៅក្រៅប្រព័ន្ធការថែទាំដែលគ្មានការបញ្ជាក់ផ្នែកវេជ្ជសាស្ត្រអំពីមូលហេតុនៃការមរណៈភាព។យើងបានធ្វើការសិក្សាតាមរយៈការធ្វើកោសល្យវិច័យដោយពាក្យសំដី ដើម្បីកំណត់មូលហេតុជាមូលដ្ឋាននៃមរណៈភាពនៅក្នុងតំបន់ជនបទនិងប៉ាន់ប្រមាណការមរណៈភាពដោយប្រើ ឆ្នាំនៃការបាត់បង់ជីវិត Year of Life Lost (YLL)

វិធីសាស្ត្រចាប់ពីឆ្នាំ២០២១-២០២៤
យើងបានធ្វើការស្ទាបស្ទង់មតិធ្វើកោសល្យវិច័យតាមប្រព័ន្ធអេឡិចត្រូនិក សម្រាប់របាយការណ៍មរណៈភាពនៅក្នុងភូមិចំនួន៥១០ នៅក្នុងប្រទេស បង់ក្លាដេស, ប្រទេសកម្ពុជា, សាធារណរដ្ឋប្រជាធិបតេយ្យ ប្រជាមានិតឡាវ,ប្រទេសមីយ៉ាន់ម៉ា និងប្រទេសថៃឡង់។ដោយប្រើប្រាស់កម្រងសំណួរការធ្វើកោសល្យវិច័យដោយពាក្យសំដីរបស់អង្គការសុខភាពពិភពលោកឆ្នាំ២០១៦, បុគ្គលិកសហគមន៍ត្រូវបានធ្វើការបណ្តុះបណ្តាលមុនពេលចុះធ្វើការសម្ភាសន៍ ហើយគ្រូពេទ្យប្រចាំតំបន់បានកំណត់មូលហេតុនៃការមរណៈភាពដោយឯករាជ្យដោយប្រើលេខកូដ ICD-10។

ការរកឃើញ លើស 90% នៃមរណៈភាពដែលបានរាយការណ៍ត្រូវបានធ្វើកោសល្យវិច័យដោយពាក្យសម្តី។ នៅក្នុងចំណោមអ្នកបានទទួលមរណៈភាព ៣៩១៣ នាក់ ៦០% គឺជាបុរស និង ៩៥% មានអាយុលើសពី១២ឆ្នាំ។ស្ទើរទូទាំងតំបន់ ៦៤-៨៦% នៃមរណៈភាពគឺកើតឡើងនៅផ្ទះ។ ជំងឺមិនឆ្លងគឺជាមូលហេតុឈានមុខនាំអោយស្លាប់, ៥៤% នៅប្រទេសឡាវ, ៧០% នៅប្រទេសកម្ពុជា ដោយជំងឺបេះដូង, សរសៃឈាមខួរក្បាល, មហារីក,និងច្រើនបំផុតគឺជំងឺផ្នែករំលាយអាហារ។ជំងឺចំពោះម្តាយ , អាហាររូបត្ថម្ភនិងជំងឺឆ្លង,ការឆ្លងមេរោគតាមផ្លូវដង្ហើម,

រាគរូស និងជំងឺរបេងមានចំនួន ១១-២៦% នៃការស្លាប់ទូទាំងតំបន់។

គ្រោះថ្នាក់ និងរបួស មានប្រមាណ ៧-១៣%និងជាប់ចំណាត់ថ្នាក់ ក្នុងចំណោមមូលហេតុចម្បងទាំងប្រាំ។គម្លាតសំខាន់ៗនៅក្នុងឯកសារមរណៈភាពនៅតែមានគ្រប់តំបន់នៅទូទាំងប្រទេសខណៈពេលដែលមូលហេតុជាក់លាក់នៃការស្លាប់ដោយជំងឺប្រែប្រួលតាមតំបន់។

ការបកស្រាយ
ការសិក្សាពហុប្រទេសបានគូសបញ្ជាក់ពីបន្ទុកដ៏សំខាន់នៃជំងឺមិនឆ្លងនៅទីជនបទក្នុងតំបន់អាស៊ីអាគ្នេយ៍, ទន្ទឹមនឹងភាពជាប់លាប់នៃជំងឺឆ្លងដោយគូសបញ្ជាក់ពីតម្រូវការធ្វើអោយប្រសើរឡើងនៃទិន្នន័យមរណៈភាព និង លទ្ធភាពទទួលបានការថែទាំសុខភាពដើម្បីកាត់បន្ថយបន្ទុកពីរនេះ។

Supplementary materials

Table of contents

Appendix 1	Selection of villages and study population..	Page 2
Appendix 2	Notification sources used for verbal autopsy interviews by study sites……...........	Page 2
Appendix 3	Cause of death with ICD-10 codes………………………………………………	Page 3
Appendix 4	Non-sex-specific life expectancy estimates by 5-Year age groups and country used in YLL Calculations ………….	Page 9
Appendix 5	Sex-Specific life expectancy estimates by 5-Year age groups and country ....…	Page 10
Appendix 6	Distribution of major cause groups…..	Page 11
Appendix 7	Sex distribution within undetermined (ill-defined) conditions group ….................	Page 11
Appendix 8	Cause-Specific Mortality Fractions for leading causes of death: A Lollipop Plot..…………..	Page 11
Appendix 9	Frequency of leading causes of death within accidents and injuries …...................	Page 12
Appendix 10A	Frequency of the top ten leading causes of deaths (number of deaths, CSMFs (%)), by Male ………………………………..	Page 12
Appendix 10B	Frequency of the top ten leading causes of deaths (number of deaths, CSMFs (%)), by Female	Page 12
Appendix 11	Crude death rate per 1,000 population, by country ……………..............................	Page 13
Appendix 12	Age and YLL distribution by major cause groups	Page 13
Appendix 13	Discrepancy, reconciliation, and adjudication rates across the sites.......................	Page 14

Appendix 1 Selection of villages and study population

The study villages were purposively chosen within the febrile illness network research program. While all selected villages were linked to primary care units or basic health facilities, these facilities do not offer expanded or enhanced services beyond what is typically available in rural areas of each country.

Inclusion criteria for villages selection within the febrile illness network are as follows.

- Villages had to be rural and underserved
- Linked to primary care units without enhanced services
- Represent diverse cultural and health system contexts
- Feasibility for long-term febrile illness diseases surveillance
- Prior collaboration with research teams

In Bangladesh, the two sites include a forcibly displaced population in Cox's Bazar and a tribal population in hilly Bandarban, both representing important and often underrepresented, rural communities. However, a few conflict-affected villages in Myanmar and Bangladesh were excluded due to restricted access.

Appendix 2 Notification sources used for verbal autopsy interviews by study sites

Country	Province/Districts	Notification sources used
Bangladesh	Cox's Bazar and Bandarban	Village health workers, local NGOs, community leaders
Cambodia	Battambang and Pailin	Commune offices, district offices, health centres
Lao PDR	Savannakhet Province (Phalanxay, Phine, Atsaphanthong)	Health centres, village chiefs
Myanmar	Hpa-An, Myawaddy, Hlaing-Bwe (Karen State)	Village leaders, malaria posts, midwives, local ethnic authorities
Thailand	Chiang Rai Province	Primary care units, village health volunteers, local administrative offices

Note: Multiple data sources were cross-checked by local teams to identify duplicates and reconcile discrepancies. Suspected omissions or disagreements were verified through community visits or calls, ensuring reliable and complete death notifications before verbal autopsy interviews.

Appendix 3 Cause of death with ICD-10 codes

Age group	Cause of death level 1	Cause of death level 2	Cause of death level 3	ICD10 Code
Adult	Category 1	Not applicable	Not applicable	A00-A99,B00-B17,B19-B99,C46,D50-D53,D64,D84,E00-E02,E40-E64,F53,G00-G09,H10,H60,H65-H68,H70-H71,I30,I32-I33,I39-I41,J00-J22,J32,J36,J65,J85-J86,K02,K04-K05,K61,K65,K67,K81,L00-L04,L08,M00-M01,M60,M86,N10,N30,N34,N41,N49,N61,N70-N74,O00-O99,P00-P96,R04,R50,R75,R95-R96,U04,U80-U89,X53-X54,Y95
Adult	Category 1	Tuberculosis	Not applicable	A15-A19,B90,J65
Adult	Category 1	Tuberculosis	Tuberculosis	A15-A19,B90,J65
Adult	Category 1	HIV/AIDS	Not applicable	B20-B24,C46,D84,R75
Adult	Category 1	HIV/AIDS	HIV/AIDS	B20-B24,C46,D84,R75
Adult	Category 1	Sexually-transmitted infections excluding HIV/AIDS	Not applicable	A51-A64,N70-N74
Adult	Category 1	Sexually-transmitted infections excluding HIV/AIDS	Syphilis	A51-A53
Adult	Category 1	Sexually-transmitted infections excluding HIV/AIDS	Other sexually transmitted infections (excluding HIV/AIDS)	A54-A64,N70-N74
Adult	Category 1	Diarrhoeal diseases	Not applicable	A00-A09
Adult	Category 1	Diarrhoeal diseases	Diarrhoeal diseases	A00-A09
Adult	Category 1	Selected vaccine preventable diseases	Not applicable	A33-A37,A80,B01,B03,B05-B06,B26,B91
Adult	Category 1	Selected vaccine preventable diseases	Tetanus	A33,A35
Adult	Category 1	Selected vaccine preventable diseases	Measles	B01,B05
Adult	Category 1	Selected vaccine preventable diseases	Poliomyelitis	A80,B91
Adult	Category 1	Selected vaccine preventable diseases	Other vaccine preventable diseases	A36-A37,B03,B06,B26
Adult	Category 1	Meningitis/encephalitis	Meningitis/encephalitis	A39,A81-A89,G00-G09
Adult	Category 1	Meningitis/encephalitis	Meningitis/encephalitis	A39,A81,A83-A89,G00-G09
Adult	Category 1	Meningitis/encephalitis	Rabies	A82
Adult	Category 1	Hepatitis	Not applicable	B15-B17,B19
Adult	Category 1	Hepatitis	Hepatitis	B15-B17,B19
Adult	Category 1	Malaria	Not applicable	B50-B54
Adult	Category 1	Malaria	Malaria	B50-B54
Adult	Category 1	Selected tropical diseases	Not applicable	A30,A71,A90-A99,B55-B58,B60-B83,B92
Adult	Category 1	Selected tropical diseases	Protozoal diseases	B55-B58,B60-B64
Adult	Category 1	Selected tropical diseases	Leprosy	A30,B92
Adult	Category 1	Selected tropical diseases	Arthropod-borne viral fevers	A90-A99
Adult	Category 1	Selected tropical diseases	Trachoma	A71
Adult	Category 1	Selected tropical diseases	Helminthiases	B65-B83
Adult	Category 1	Respiratory infections	Not applicable	H65-H68,H70-H71,J00-J22,J32,J36,J85-J86,P23,U04

Adult	Category 1	Respiratory infections	Acute respiratory infections	H65-H68,H70-H71,J00-J22,J32,J36,J85-J86,P23,U04
Adult	Category 1	Acute bacterial sepsis & severe Infections	Not applicable	A20-A28,A32,A38,A40-A49,A68,A70,A74-A79,B95-B96,H10,H60,I30,I32-I33,I39-I41,K02,K04-K05,K61,K65,K67,K81,L00-L04,L08,M00-M01,M60,M86,N10,N30,N34,N41,N49,N61,P36-P39,U80-U89
Adult	Category 1	Acute bacterial sepsis & severe Infections	Severe Systemic Infection	A20-A28,A32,A38,A40-A49,A68,A70,A74-A79,B95-B96,P36-P39,U80-U89
Adult	Category 1	Acute bacterial sepsis & severe Infections	Severe Localized Infection	H10,H60,I30,I32-I33,I39-I41,K02,K04-K05,K61,K65,K67,K81,L00-L04,L08,M00-M01,M60,M86,N10,N30,N34,N41,N49,N61
Adult	Category 1	Other infectious and parasitic diseases	Not applicable	A31,A50,A65-A67,A69,B00,B02,B04,B07-B09,B25,B27-B49,B59,B85-B89,B94,B97-B99,P35,Y95
Adult	Category 1	Other infectious and parasitic diseases	Other infectious diseases	A31,A50,A65-A67,A69,B00,B02,B04,B07-B09,B25,B27-B49,B59,B85-B89,B94,B97-B99,P35,Y95
Adult	Category 1	Maternal conditions	Not applicable	O00-O99,A34,F53
Adult	Category 1	Maternal conditions	Abortion or miscarriage	O00-O08
Adult	Category 1	Maternal conditions	Hypertensive disorders of pregnancy	O11-O16
Adult	Category 1	Maternal conditions	Obstetric haemorrhage	O20,O43-O46,O67,O71-O72
Adult	Category 1	Maternal conditions	Maternal sepsis	O23,O41,O85-O86,O91,A34
Adult	Category 1	Maternal conditions	Other maternal conditions	O10,O21-O22,O24-O40,O42,O47-O66,O68-O70,O73-O84,O87-O90,O92-O99,F53
Adult	Category 1	Perinatal conditions	Not applicable	P00-P22,P24-P29,P50-P96,R95,R96
Adult	Category 1	Perinatal conditions	Low birth weight/preterm	P01,P05-P07,P22,P25-P28,P52,P61,P77
Adult	Category 1	Perinatal conditions	Birth asphyxia and birth trauma	P02-P03,P10-P15,P20-P21,P24,P50,P90-P91
Adult	Category 1	Perinatal conditions	Other perinatal conditions	P00,P04,P08,P29,P51,P53-P60,P70-P76,P78-P83,P92-P94,P96,R95,R96
Adult	Category 1	Nutritional deficiencies	Not applicable	D50-D53,D64,E00-E02,E40-E46,E50-E64,X53-X54
Adult	Category 1	Nutritional deficiencies	Protein-energy malnutrition	E40-E46,X53-X54
Adult	Category 1	Nutritional deficiencies	Iron, vitamin deficiencies and nutritional anemias	D50-D53,D64,E00-E02,E50-E64
Adult	Category 1	Fever of unknown origin	Not applicable	R50
Adult	Category 1	Fever of unknown origin	Fever of unknown origin	R50
Adult	Category 2	Not applicable	Not applicable	B18,C00-C45,C47-C97,D00-D48,D55-D63,D65-D83,D86-D89,E03-E35,E65-E90,F00-F52,F54-F99,G10-G99,H00-H06,H11-H59,H61-H62,H69,H72-H95,I00-I28,I31,I34-I38,I42-I99,J30-J31,J33-J35,J37-J64,J66-J84,J90-J99,K00-K01,K03,K06-K60,K62-K63,K66,K70-K80,K82-K93,L05,L10-L99,M02-M54,M61-M85,M87-M99,N00-N08,N11-N29,N31-N33,N35-N40,N42-N48,N50-N60,N62-N64,N75-N99,Q00-Q99,R00-R01,R03,R05-R06,R10-R23,R26-R49,R55-R56,R59,R63,R70-R74,R76-R77,R80-R82,R84-R87,R90-R91,R96,X45,Y15,Y90-Y91
Adult	Category 2	Neoplasms	Not applicable	C00-C45,C47-C97,D00-D48,N60,N62-N64,N87,R59
Adult	Category 2	Neoplasms	Upper aerodigestive	C00-C15,C30-C32,D10-D11
Adult	Category 2	Neoplasms	Stomach	C16
Adult	Category 2	Neoplasms	Colon, rectum and small intestine	C17-C21,D12
Adult	Category 2	Neoplasms	Liver	C22
Adult	Category 2	Neoplasms	Other digestive	C23-C26,C48,D00-D01,D13,D37
Adult	Category 2	Neoplasms	Lung & airway cancers	C33-C34,C39,D02,D14
Adult	Category 2	Neoplasms	Breast cancer	C50,D05,D24,N60,N62-N64
Adult	Category 2	Neoplasms	Cervix & Uterus cancers	C53-C55,D06,N87
Adult	Category 2	Neoplasms	Other female genital cancer	C51-C52,C56-C58,D25-D28,D39

Adult	Category 2	Neoplasms	Male genital cancers	C60-C63,D29,D40
Adult	Category 2	Neoplasms	Urinary	C64-C68,D30,D41
Adult	Category 2	Neoplasms	Brain and eye	C69-C72,D31-D33,D42-D43
Adult	Category 2	Neoplasms	Leukemias, lymphomas and other hematopoietic malignancies	C81-C96,D45-D47
Adult	Category 2	Neoplasms	Other Neoplasms	C37-C38,C40-45,C47,C49,C73-C75,C77-C79,C97,D03-D04,D07-D09,D15-D23,D34-D36,D38,D44,R59
Adult	Category 2	Neoplasms	Cancer with unspecified sites	C76,C80,D48
Adult	Category 2	Diabetes mellitus	Not applicable	E10-E14
Adult	Category 2	Diabetes mellitus	Diabetes mellitus	E10-E14
Adult	Category 2	Endocrine and immune disorders	Not applicable	D55-D63,D65-D83,D86-D89,E03-E07,E15-E16,E20-E35,E65-E90,R70-R74,R76-R77,R81
Adult	Category 2	Endocrine and immune disorders	Endocrine and immune disorders	D55-D63,D65-D83,D86-D89,E03-E07,E15-E16,E20-E35,E65-E90,R70-R74,R76-R77,R81
Adult	Category 2	Neuro-psychiatric conditions	Not applicable	F00-F09,F11-F52,F54-F99,G10-G44,G47-G80,G90-G99,R26-R29,R40-R49,R56,R90
Adult	Category 2	Neuro-psychiatric conditions	Epilepsy	G40-G41,R56
Adult	Category 2	Neuro-psychiatric conditions	Other neuropsychiatric disorders	F00-F09,F11-F39,F40-F52,F54-F99,G10-G37,G43-G44,G47-G73,G80,G90-G99,R26-R29,R40-R49,R90
Adult	Category 2	Skin, Musculoskeletal, Sense Organ, Oral disorders	Not applicable	H00-H06,H11-H59,H61-H62,H69,H72-H95,K00-K01,K03,K06-K14,L05,L10-L99,M02-M54,M61-M85,M87-M99,R20-R23
Adult	Category 2	Skin, Musculoskeletal, Sense Organ, Oral disorders	Skin diseases	L05,L10-L99,R20-R23
Adult	Category 2	Skin, Musculoskeletal, Sense Organ, Oral disorders	Musculoskeletal disorders	M02-M54,M61-M85,M87-M99
Adult	Category 2	Skin, Musculoskeletal, Sense Organ, Oral disorders	Sense organ disorders	H00-H06,H11-H59,H61-H62,H69,H72-H95
Adult	Category 2	Skin, Musculoskeletal, Sense Organ, Oral disorders	Oral conditions	K00-K01,K03,K06-K14
Adult	Category 2	Cardiovascular diseases	Not applicable	G45-G46,G81-G83,I00-I28,I31,I34-I38,I42-I84,I86-I99,R00-R01,R03,R55,R96
Adult	Category 2	Cardiovascular diseases	Rheumatic heart disease	I00-I09,I38
Adult	Category 2	Cardiovascular diseases	Hypertensive heart diseases	I10-I15
Adult	Category 2	Cardiovascular diseases	Ischaemic heart diseases	I20-I25,I44,I46,I70,R55,R96
Adult	Category 2	Cardiovascular diseases	Cerebrovascular disease	I60-I69,G45-G46,G81-G83
Adult	Category 2	Cardiovascular diseases	Heart failure	I50
Adult	Category 2	Cardiovascular diseases	Other cardiovascular diseases	I26-I28,I31,I34-I37,I42-I43,I45,I47-I49,I51-I52,I71-I84,I86-I99,R00-R01,R03
Adult	Category 2	Chronic Respiratory diseases	Not applicable	J30-J31,J33-J35,J37-J64,J66-J84,J90-J99,R04-R06,R84,R91
Adult	Category 2	Chronic Respiratory diseases	Asthma and Chronic obstructive pulmonary disease	J40-J47,J63,J93
Adult	Category 2	Chronic Respiratory diseases	Other chronic respiratory diseases	J30-J31,J33-J35,J37-J39,J60-J62,J64,J66-J84,J90-J92,J94-J99,R04-R06,R84,R91
Adult	Category 2	Digestive diseases	Not applicable	B18,F10,I85,K20-K60,K62-K63,K66,K70-K80,K82-K93,R10-R19,R63,R85,X45,Y15,Y90,Y91
Adult	Category 2	Digestive diseases	Gastro-oesophageal	K20-K23,K25-K31,I85,R12,R13
Adult	Category 2	Digestive diseases	Liver and alcohol related diseases	B18,F10,K70-K77,R16-R18,X45,Y15,Y90-Y91
Adult	Category 2	Digestive diseases	Other digestive diseases	K35-K38,K40-K60,K62-K63,K66,K80,K82-K93,R10-R11,R14-R15,R19,R63,R85

Adult	Category 2	Genito-urinary diseases	Not applicable	N00-N08,N11-N29,N31-N33,N35-N40,N42-N48,N50-N51,N75-N86,N88-N99,R30-R39,R80,R82,R86,R87
Adult	Category 2	Genito-urinary diseases	Nephritis and nephrosis	N00-N08,N11-N19
Adult	Category 2	Genito-urinary diseases	Other genitourinary system diseases	N20-N29,N31-N33,N35-N40,N42-N48,N50-N51,N75-N86,N88-N99,R30-R39,R80,R82,R86,R87
Adult	Category 2	Congenital anomalies	Not applicable	Q00-Q99
Adult	Category 2	Congenital anomalies	Congenital anomalies	Q00-Q99
Adult	Category 3	Not applicable	Not applicable	V01-V99,W00-W99,X00-X44,X46-X52,X57-Y14,Y16-Y36,Y40-Y89,Y96-Y98
Adult	Category 3	Unintentional Category 3	Not applicable	V01-V99,W00-W99,X00-X44,X46-X52,X57-X59,Y40-Y86,Y88-Y89
Adult	Category 3	Unintentional Category 3	Road traffic accidents	V01-V89,V99,Y85
Adult	Category 3	Unintentional Category 3	Other transport accidents	V90-V98
Adult	Category 3	Unintentional Category 3	Poisonings	X40-X44,X46-X49
Adult	Category 3	Unintentional Category 3	Falls	W00-W19
Adult	Category 3	Unintentional Category 3	Fires	X00-X09
Adult	Category 3	Unintentional Category 3	Drownings	W65-W74
Adult	Category 3	Unintentional Category 3	Venomous snakes, animals and plants	X20-X29,W57,W60
Adult	Category 3	Unintentional Category 3	Other unintentional Category 3	W20-W56,W58-W59,W64,W75-W99,X10-X19,X30-X39,X50-X52,X57-X59,Y40-Y84,Y86,Y88-Y89
Adult	Category 3	Intentional Category 3	Not applicable	X60-Y09,Y35-Y36,Y87
Adult	Category 3	Intentional Category 3	Self-inflicted Category 3 (suicide)	X60-X84
Adult	Category 3	Intentional Category 3	Interpersonal violence and other intentional Category 3	X85-X99,Y00-Y09,Y35-Y36,Y87
Adult	Category 3	Undetermined Intent	Not applicable	Y10-Y14,Y16-Y34,Y96-Y98
Adult	Category 3	Undetermined Intent	Undetermined Intent	Y10-Y14,Y16-Y34,Y96-Y98
Adult	Category 3	Undetermined Intent	Injury and poisoning -* MDS instruction given Not to code this category	S00-S99,T00-T98
Adult	Category 4	Undetermined Intent	Not applicable	R02,R07-R09,R25,R51-R54,R57-R58,R60-R62,R64-R69,R78-R79,R83,R89,R92-R94,R96,R98-R99
Adult	Category 4	Ill-defined or cause unknown	Not applicable	R02,R07-R09,R25,R51-R54,R57-R58,R60-R62,R64-R69,R78-R79,R83,R89,R92-R94,R96,R98-R99
Adult	Category 4	Ill-defined or cause unknown	Senility	R54
Adult	Category 4	Ill-defined or cause unknown	Other Ill-defined and abnormal findings	R02,R07-R09,R25,R51-R53,R57,R58,R60-R62,R64-R69,R78-R79,R83,R89,R92-R94,R96,R98
Adult	Category 4	Ill-defined or cause unknown	Unspecified deaths	R99
Adult	Factors influencing health status and contact with health services	Ill-defined or cause unknown	Not applicable	Z00-Z99
Adult	Factors influencing health status and contact with health services	Factors influencing health status and contact with health services (MDS not implemented this category)	Not applicable	Z00-Z99

Adult	Factors influencing health status and contact with health services	Factors influencing health status and contact with health services (MDS not implemented this category)	Factors influencing health status (MDS not implemented this category)	Z00-Z99
Child	Category 1	Not applicable	Not applicable	A39,A81-A89,G00-G09,A20-A28,A32,A38,A40-A44,A46,A48,A49,A68-A70,A74,A75,A77-A79,B95,B96,H10,H60,I30,I32,I33,I39-I41,K65,K67,K81,L00-L04,L08,M00-M01,M60,M86,N10,N30,N34,N41,N49,N61,P36,P38,A37,H65-H68,H70,H71,J00-J22,J32,J36,J85,J86,P23,U04,R50,A30,A31,A36,A50-A67,A71,A90-A99,B00,B02-B04,B06-B09,B15-B19,B25-B27,B30,B33-B49,B55-B60,B64-B83,B85-B89,B92,B94,B97,B99,K02,K04,K05,K61,N70-N74,P35,P37,P39,U00,Y95,B50-B54,B20-B24,R75,B01,B05,A80,B91,A33-A35,A15-A19,B90,J65,A00-A09,P00,P02,P03.P10-P15,P20,P21,P24,P29,P50,P90,P91,P01,P05,P07,P22,P25-P28,P52,P61,P77,Q00-Q07,Q10-18,Q20-Q28,Q30-Q45,Q50-56,Q60-Q87,Q89-Q93,Q95,Q96-Q99,D50-D53,E00-E02,E40-E46,E50-E56,E59-E61,E63,E64,X53-X54
Child	Category 1	Early Childhood infections	Not applicable	A39,A81-A89,G00-G09,A20-A28,A32,A38,A40-A44,A46,A48,A49,A68-A70,A74,A75,A77-A79,B95,B96,H10,H60,I30,I32,I33,I39-I41,K65,K67,K81,L00-L04,L08,M00-M01,M60,M86,N10,N30,N34,N41,N49,N61,P36,P38,A37,H65-H68,H70,H71,J00-J22,J32,J36,J85,J86,P23,U04
Child	Category 1	Early Childhood infections	Pneumonia	A37,H65-H68,H70,H71,J00-J22,J32,J36,J85,J86,P23,U04
Child	Category 1	Early Childhood infections	Acute bacterial sepsis and severe infections	A20-A28,A32,A38,A40-A44,A46,A48,A49,A68-A70,A74,A75,A77-A79,B95,B96,H10,H60,I30,I32,I33,I39-I41,K65,K67,K81,L00-L04,L08,M00-M01,M60,M86,N10,N30,N34,N41,N49,N61,P36,P38
Child	Category 1	Early Childhood infections	Meningitis/encephalitis	A39,A81-A89,G00-G09
Child	Category 1	Other communicable diseases	Not applicable	R50,A30,A31,A36,A50-A67,A71,A90-A99,B00,B02-B04,B06-B09,B15-B19,B25-B27,B30,B33-B49,B55-B60,B64-B83,B85-B89,B92,B94,B97,B99,K02,K04,K05,K61,N70-N74,P35,P37,P39,U00,Y95,B50-B54,B20-B24,R75,B01,B05,A80,B91,A33-A35,A15-A19,B90,J65,A00-A09
Child	Category 1	Other communicable diseases	Diarrhoeal diseases	A00-A09
Child	Category 1	Other communicable diseases	Tuberculosis	A15-A19,B90,J65
Child	Category 1	Other communicable diseases	Tetanus	A33-A35
Child	Category 1	Other communicable diseases	Poliomyelitis	A80,B91
Child	Category 1	Other communicable diseases	Measles	B01,B05
Child	Category 1	Other communicable diseases	HIV/AIDS	B20-B24,R75
Child	Category 1	Other communicable diseases	Malaria	B50-B54
Child	Category 1	Other communicable diseases	Other infectious and parasitic diseases	A30,A31,A36,A50-A67,A71,A90-A99,B00,B02-B04,B06-B09,B15-B19,B25-B27,B30,B33-B49,B55-B60,B64-B83,B85-B89,B92,B94,B97,B99,K02,K04,K05,K61,N70-N74,P35,P37,P39,U00,Y95
Child	Category 1	Other communicable diseases	Fever of unknown origin	R50
Child	Category 1	Perinatal conditions	Not applicable	P00,P02,P03.P10-P15,P20,P21,P24,P29,P50,P90,P91,P01,P05,P07,P22,P25-P28,P52,P61,P77
Child	Category 1	Perinatal conditions	Prematurity & low birthweight	P01,P05,P07,P22,P25-P28,P52,P61,P77
Child	Category 1	Perinatal conditions	Birth asphyxia & birth trauma	P00,P02,P03.P10-P15,P20,P21,P24,P29,P50,P90,P91
Child	Category 1	Other conditions	Not applicable	Q00-Q07,Q10-18,Q20-Q28,Q30-Q45,Q50-56,Q60-Q87,Q89-Q93,Q95,Q96-Q99,D50-D53,E00-E02,E40-E46,E50-E56,E59-E61,E63,E64,X53-X54
Child	Category 1	Other conditions	Nutritional diseases	D50-D53,E00-E02,E40-E46,E50-E56,E59-E61,E63,E64,X53-X54
Child	Category 1	Other conditions	Congenital anomalies	Q00-Q07,Q10-18,Q20-Q28,Q30-Q45,Q50-56,Q60-Q87,Q89-Q93,Q95,Q96-Q99

Child	Category 2	Not applicable	Not applicable	C00-C97,D01-D48,D55-D89,E03-E35,E65-E90,F00-F99,G10-G99,H00-H06,H11-H59,H61-H62,H69,H72-H95,I00-I28,I31,I34-I38,I42-I99,J30,J31,J33-J35,J37-J47,J60,J64,J66-J70,J80-J82,J84,J90-J99,K00-K01,K03,K06-K60,K62-K63,K70-K80,K82-K93,L05,L10-L99,M02-M54,M61-M85,M87-M99,N00-N08,N11-N29,N31-N33,N35-N40,N42-N48,N50-N51,N60,N62-N64,N75-N99,P04,P08,P51,P53-P60,P70-P72,P74-P76,P78,P80-P83,P92-P94,R00,R01,R03-R05,R06,R11-R23,R26,R27,R29-R49,R55-R56,R59,R63,R70-R74,R76-R77,R80-R82,R84-R87,R90-R91
Child	Category 2	Other Category 2	Not applicable	C00-C97,D01-D48,D55-D89,E03-E35,E65-E90,F00-F99,G10-G99,H00-H06,H11-H59,H61-H62,H69,H72-H95,I00-I28,I31,I34-I38,I42-I99,J30,J31,J33-J35,J37-J47,J60,J64,J66-J70,J80-J82,J84,J90-J99,K00-K01,K03,K06-K60,K62-K63,K70-K80,K82-K93,L05,L10-L99,M02-M54,M61-M85,M87-M99,N00-N08,N11-N29,N31-N33,N35-N40,N42-N48,N50-N51,N60,N62-N64,N75-N99,P04,P08,P51,P53-P60,P70-P72,P74-P76,P78,P80-P83,P92-P94,R00,R01,R03-R05,R06,R11-R23,R26,R27,R29-R49,R55-R56,R59,R63,R70-R74,R76-R77,R80-R82,R84-R87,R90-R91
Child	Category 2	Other Category 2	Other Category 2	C00-C97,D01-D48,D55-D89,E03-E35,E65-E90,F00-F99,G10-G99,H00-H06,H11-H59,H61-H62,H69,H72-H95,I00-I28,I31,I34-I38,I42-I99,J30,J31,J33-J35,J37-J47,J60,J64,J66-J70,J80-J82,J84,J90-J99,K00-K01,K03,K06-K60,K62-K63,K70-K80,K82-K93,L05,L10-L99,M02-M54,M61-M85,M87-M99,N00-N08,N11-N29,N31-N33,N35-N40,N42-N48,N50-N51,N60,N62-N64,N75-N99,P04,P08,P51,P53-P60,P70-P72,P74-P76,P78,P80-P83,P92-P94,R00,R01,R03-R05,R06,R11-R23,R26,R27,R29-R49,R55-R56,R59,R63,R70-R74,R76-R77,R80-R82,R84-R87,R90-R91
Child	Category 3	Not applicable	Not applicable	S00-S99,T00-T98,V01-V99,W00-W99,X00-X52,X57-X99,Y00-Y91,Y97-Y98.
Child	Category 3	Category 3	Not applicable	S00-S99,T00-T98,V01-V99,W00-W99,X00-X52,X57-X99,Y00-Y91,Y97-Y98.
Child	Category 3	Category 3	Category 3	S00-S99,T00-T98,V01-V99,W00-W99,X00-X52,X57-X99,Y00-Y91,Y97-Y98.
Child	Category 4	Not applicable	Not applicable	P96,R02,R07,R09,R10,R25,R51-R54,R57-R58,R60-R62,R64,R68,R69,R78,R79,R83,R89,R92-R99
Child	Category 4	Ill-defined or cause unknown	Not applicable	P96,R02,R07,R09,R10,R25,R51-R54,R57-R58,R60-R62,R64,R68,R69,R78,R79,R83,R89,R92-R99
Child	Category 4	Ill-defined or cause unknown	Ill-defined or cause unknown	P96,R02,R07,R09,R10,R25,R51-R54,R57-R58,R60-R62,R64,R68,R69,R78,R79,R83,R89,R92-R99
Neonate	Category 1	Not applicable	Not applicable	A37,H65-H68,H70,H71,J00-J22,J32,J36,J85,J86,P23,U04,A20-A28,A32,A38,A40-A44,A46,A48,A49,A68-A70,A74,A75,A77-A79,B95,B96,H10,H60,I30,I32-I33,I39-I41,K65,K67,K81,L00-L04,L08,M00-M01,M60,M86,N10,N30,N34,N41,N49,N61,P35-P39,A39,A81-A89,G00-G09,A00-A09,A33-A35,A80,B91,B01,B05,B50-B54,A15-A19,A30-A31,A36,A50-A67,A71,A90-A99,B00,B02-B04,B06-B09,B15-B27,B30,B33-B49,B55-B60,B64-B83,B85-B90,B92,B94,B97,B99,J65,K04,K05,K61,N70-N74,R75,U00,Y95,R50,P01,P05,P07,P22,P25-P28,P52,P61,P77,R04,P00,P02,P03,P10-P15,P20,P21,P24,P29,P50,P90,P91,G10-G99,Q00-Q99,D50-D53,E00-E02,E40-E46,E50-E56,E59-E61,E63,E64,X53-X54
Neonate	Category 1	Neonatal Infections	Not applicable	A37,H65-H68,H70,H71,J00-J22,J32,J36,J85,J86,P23,U04,A20-A28,A32,A38,A40-A44,A46,A48,A49,A68-A70,A74,A75,A77-A79,B95,B96,H10,H60,I30,I32-I33,I39-I41,K65,K67,K81,L00-L04,L08,M00-M01,M60,M86,N10,N30,N34,N41,N49,N61,P35-P39,A39,A81-A89,G00-G09
Neonate	Category 1	Neonatal Infections	Neonatal Pneumonia	A37,H65-H68,H70,H71,J00-J22,J32,J36,J85,J86,P23,U04
Neonate	Category 1	Neonatal Infections	Sepsis	A20-A28,A32,A38,A40-A44,A46,A48,A49,A68-A70,A74,A75,A77-A79,B95,B96,H10,H60,I30,I32-I33,I39-I41,K65,K67,K81,L00-L04,L08,M00-M01,M60,M86,N10,N30,N34,N41,N49,N61,P35-P39
Neonate	Category 1	Neonatal Infections	Meningitis/encephalitis	A39,A81-A89,G00-G09
Neonate	Category 1	Other communicable diseases	Not applicable	A00-A09,A33-A35,A80,B91,B01,B05,B50-B54,A15-A19,A30-A31,A36,A50-A67,A71,A90-A99,B00,B02-B04,B06-B09,B15-B27,B30,B33-B49,B55-B60,B64-B83,B85-B90,B92,B94,B97,B99,J65,K04,K05,K61,N70-N74,R75,U00,Y95,R50
Neonate	Category 1	Other communicable diseases	Diarrhoeal diseases	A00-A09

Neonate	Category 1	Other communicable diseases	Tetanus	A33-A35
Neonate	Category 1	Other communicable diseases	Poliomyelitis	A80,B91
Neonate	Category 1	Other communicable diseases	Measles	B01,B05
Neonate	Category 1	Other communicable diseases	Malaria	B50-B54
Neonate	Category 1	Other communicable diseases	Other infectious and parasitic diseases	A15-A19,A30-A31,A36,A50-A67,A71,A90-A99,B00,B02-B04,B06-B09,B15-B27,B30,B33-B49,B55-B60,B64-B83,B85-B90,B92,B94,B97,B99,J65,K04,K05,K61,N70-N74,R75,U00,Y95
Neonate	Category 1	Other communicable diseases	Fever of unknown origin	R50
Neonate	Category 1	Perinatal conditions	Not applicable	P01,P05,P07,P22,P25-P28,P52,P61,P77,R04,P00,P02,P03,P10-P15,P20,P21,P24,P29,P50,P90,P91
Neonate	Category 1	Perinatal conditions	Prematurity & low birthweight	P01,P05,P07,P22,P25-P28,P52,P61,P77,R04
Neonate	Category 1	Perinatal conditions	Birth asphyxia & birth trauma	P00,P02,P03,P10-P15,P20,P21,P24,P29,P50,P90,P91
Neonate	Category 1	Other conditions	Not applicable	G10-G99,Q00-Q99,D50-D53,E00-E02,E40-E46,E50-E56,E59-E61,E63,E64,X53-X54
Neonate	Category 1	Other conditions	Congenital anomalies	G10-G99,Q00-Q99
Neonate	Category 1	Other conditions	Nutritional diseases	D50-D53,E00-E02,E40-E46,E50-E56,E59-E61,E63,E64,X53-X54
Neonate	Category 2	Not applicable	Not applicable	C00-C97,D01-D48,D55-D89,E03-E35,E65-E90,F00-F99,H00-H06,H11-H59,H61-H62,H69,H72-H95,I00-I28,I31,I34-I38,I42-I99,J30,J31,J33-J35,J37-J47,J60,J64,J66-J70,J80-J82,J84,J90-J99,K00-K03,K06-K60,K62-K63,K70-K80,K82-K93,L05,L10-L99,M02-M54,M61-M85,M87-M99,N00-N08,N11-N29,N31-N33,N35-N40,N42-N48,N50-N51,N60,N62-N64,N75-N99,P04,P08,P51,P53-P60,P70-P72,P74-P76,P78,P80-P83,P92-P94,R00,R01,R03,R05,R06,R11-R23,R26,R27,R29-R36,R39-R49,R55,R56,R59,R63,R70-R74,R76-R77,R80-R82,R84-R87,R90,R91
Neonate	Category 2	Other Category 2	Not applicable	C00-C97,D01-D48,D55-D89,E03-E35,E65-E90,F00-F99,H00-H06,H11-H59,H61-H62,H69,H72-H95,I00-I28,I31,I34-I38,I42-I99,J30,J31,J33-J35,J37-J47,J60,J64,J66-J70,J80-J82,J84,J90-J99,K00-K03,K06-K60,K62-K63,K70-K80,K82-K93,L05,L10-L99,M02-M54,M61-M85,M87-M99,N00-N08,N11-N29,N31-N33,N35-N40,N42-N48,N50-N51,N60,N62-N64,N75-N99,P04,P08,P51,P53-P60,P70-P72,P74-P76,P78,P80-P83,P92-P94,R00,R01,R03,R05,R06,R11-R23,R26,R27,R29-R36,R39-R49,R55,R56,R59,R63,R70-R74,R76-R77,R80-R82,R84-R87,R90,R91
Neonate	Category 2	Other Category 2	Other Category 2	C00-C97,D01-D48,D55-D89,E03-E35,E65-E90,F00-F99,H00-H06,H11-H59,H61-H62,H69,H72-H95,I00-I28,I31,I34-I38,I42-I99,J30,J31,J33-J35,J37-J47,J60,J64,J66-J70,J80-J82,J84,J90-J99,K00-K03,K06-K60,K62-K63,K70-K80,K82-K93,L05,L10-L99,M02-M54,M61-M85,M87-M99,N00-N08,N11-N29,N31-N33,N35-N40,N42-N48,N50-N51,N60,N62-N64,N75-N99,P04,P08,P51,P53-P60,P70-P72,P74-P76,P78,P80-P83,P92-P94,R00,R01,R03,R05,R06,R11-R23,R26,R27,R29-R36,R39-R49,R55,R56,R59,R63,R70-R74,R76-R77,R80-R82,R84-R87,R90,R91
Neonate	Category 3	Not applicable	Not applicable	S00-S99,T00-T98,V01-V99,W00-W99,X00-X52,X57-X99,Y00-Y91,Y97-Y98
Neonate	Category 3	Category 3	Not applicable	S00-S99,T00-T98,V01-V99,W00-W99,X00-X52,X57-X99,Y00-Y91,Y97-Y98
Neonate	Category 3	Category 3	Category 3	S00-S99,T00-T98,V01-V99,W00-W99,X00-X52,X57-X99,Y00-Y91,Y97-Y98
Neonate	Category 4	Not applicable	Not applicable	P96,R02,R07,R09,R10,R25,R51-R54,R57-R58,R60-R62,R64,R68,R69,R78,R79,R83,R89,R92-R99
Neonate	Category 4	Ill-defined or cause unknown	Not applicable	P96,R02,R07,R09,R10,R25,R51-R54,R57-R58,R60-R62,R64,R68,R69,R78,R79,R83,R89,R92-R99
Neonate	Category 4	Ill-defined or cause unknown	Ill-defined or cause unknown	P96,R02,R07,R09,R10,R25,R51-R54,R57-R58,R60-R62,R64,R68,R69,R78,R79,R83,R89,R92-R99

Appendix 4 Non-sex-specific life expectancy estimates by 5-Year age groups and country used in YLL Calculations[17]

Age groups (years)	Bangladesh	Myanmar	Thailand	Lao PDR	Cambodia
0-4	74	66	77	68	69
5-9	69	61	72	64	64
10-14	64	57	67	59	59

15-19	58	52	62	54	55
20-24	54	47	57	49	50
25-29	49	43	52	45	46
30-34	43	38	48	40	41
35-39	40	34	43	35	37
40-44	35	30	39	31	32
45-49	30	25	34	27	28
50-54	26	21	30	22	24
55-59	22	18	26	18	20
60-64	18	14	22	15	16
65-69	16	11	18	12	13
70-74	12	8	15	9	10
75-79	9	6	12	7	7
80-84	7	4	10	5	5
85-89	5	3	8	3	4
90-94	3	2	6	2	3
>95	2	2	5	2	2

Appendix 5 Sex-Specific life expectancy estimates by 5-Year age groups and country [17]

Age groups (years)	Bangladesh (M, F)	Myanmar (M, F)	Thailand (M, F)	Lao PDR (M, F)	Cambodia (M, F)
0-4	73,76	64,70	70,79	67,71	67,72
5-9	68,71	59,65	65,74	62,66	63,67
10-14	63,66	55,61	61,69	58,62	58,63
15-19	58,61	50,56	56,64	53,57	53,58
20-24	54,57	46,51	51,59	49,52	48,53
25-29	49,52	41,46	47,55	44,47	45,49
30-34	44,47	37,42	42,50	39,43	40,44
35-39	39,42	33,37	38,45	35,38	36,39
40-44	35, 37	29,33	34,40	30,33	32,35
45-49	30,33	25,28	30,36	26,29	28,30
50-54	26,28	21,24	27,32	22,25	24,26
55-59	22,24	17,20	23,27	18,20	20,22
60-64	18,20	14,16	20,23	14,16	16,18
65-69	15,17	11,13	16,19	11,13	13,14
70-74	12,13	8,10	13,15	9,10	10,11
75-79	9,10	6,7	10,12	6,7	7,8
80-84	7,8	4,5	8,9	5,5	5,6
85-89	5,6	3,3	6,6	3,4	3,4
90-94	3,4	2,2	4,5	2,3	3,3
>95	2,3	2,2	3,3	2,2	2,2

M male; F female; Each cell shows the life expectancies of male and female for each age group

Appendix 6 Distribution of major cause groups (N=3,413)

Major cause groups	Bangladesh		Myanmar		Thailand		Lao PDR		Cambodia	
	Male	Female	Male	Female	Male	Female	Male	Female	Male	Female
Communicable, maternal, perinatal and nutritional causes	49	45	23	13	84	52	243	138	15	9
Non-communicable	209	135	113	77	358	244	461	342	92	63
Accidents and injuries	25	12	30	8	72	44	78	20	20	4
Ill-defined conditions	17	18	15	17	22	18	120	90	6	12

Appendix 7 Sex distribution within undetermined (ill-defined) conditions (N=335)

Undetermined conditions	Bangladesh		Myanmar		Thailand		Lao PDR		Cambodia	
	Male	Female	Male	Female	Male	Female	Male	Female	Male	Female
Senility (70 years and above)	11	11	8	15	18	17	57	73	5	11
Ill-defined	6	7	7	2	4	1	63	17	1	1

Appendix 8 Cause-Specific Mortality Fractions for leading causes of death: A Lollipop Plot

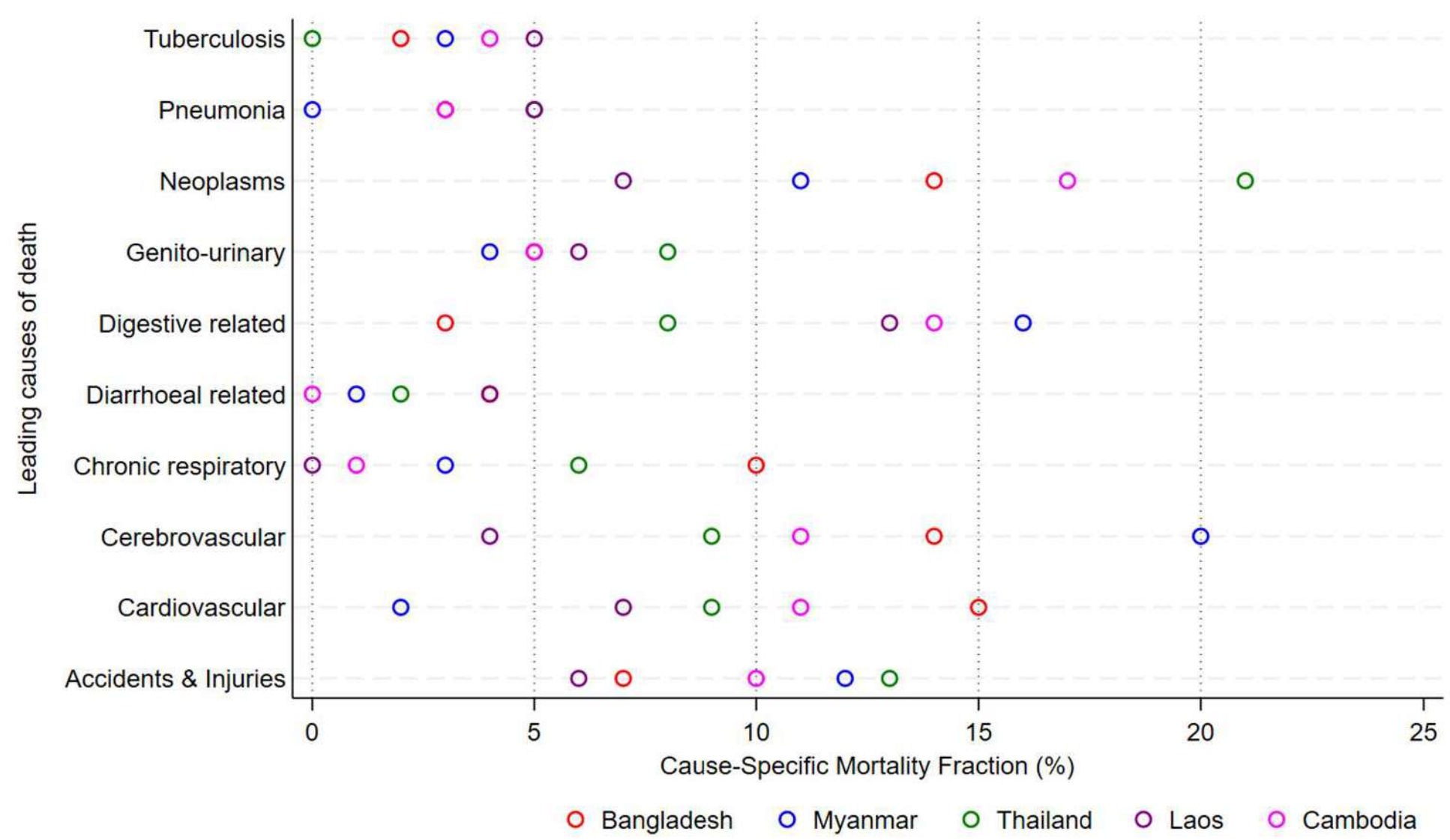

Appendix 9 Frequency of leading causes of death within accidents and injuries group, N= 313, (n, %)

Rank	Bangladesh	Myanmar	Thailand	Lao PDR	Cambodia
1	Falls (9, 24%)	Suicides (14,37%)	Road traffic injuries (39, 34%)	Road traffic injuries (31, 32%)	Road traffic injuries (11, 46%)
2	Road traffic injuries (6, 16%)	Road traffic injuries (7, 18%)	Falls (38, 33%)	Suicides (26, 27%)	Suicides (6, 25%)
3	Drowning (5, 14%)	Drowning (3, 8%)	Suicides (17, 15%)	Drowning (17, 17%)	Falls (2, 8%)

Appendix 10A Frequency of the top ten leading causes of deaths (number of deaths, CSMFs (%)), by Male

Bangladesh	No	%	Myanmar	No	%	Thailand	No	%	Lao PDR	No	%	Cambodia	No	%
Cardiovascular	50	17	Cerebrovascular	36	20	Neoplasms	117	13	Digestive related	133	15	Accidents and injuries	20	15
Cerebrovascular	44	15	Digestive related	34	19	Accidents and injuries	72	8	Accidents and injuries	79	9	Neoplasms	18	14
Neoplasms	45	15	Accidents and injuries	30	17	Digestive related	54	6	Cardiovascular	76	8	Cerebrovascular	18	14
Chronic respiratory diseases	23	11	Neoplasms	19	10	Cerebrovascular	52	6	Neoplasms	56	6	Digestive related	16	12
Accidents & Injuries	25	8	Genito-urinary related	7	4	Genito-urinary related	42	5	Tuberculosis	54	6	Cardiovascular	15	11
Genito-urinary related	17	6	Tuberculosis	6	3	Pneumonia	41	5	Pneumonia	50	6	Genito-urinary	10	8
Diarrhoeal related	6	2	Diabetes	5	3	Cardiovascular	39	4	Genito-urinary	51	6	Diabetes	9	7
Digestive related	17	6	Chronic respiratory diseases	4	2	Chronic respiratory diseases	33	4	Diarrhoeal related	48	5	Pneumonia	7	5
Pneumonia	13	4	Cardiovascular	2	1	Diabetes	13	1	Diabetes	44	5	Tuberculosis	6	5
Tuberculosis	7	2	Diarrhoea related	1	1	Sepsis	13	1	Cerebrovascular	39	4	Chronic respiratory diseases	3	2

Appendix 10B Frequency of the top ten leading causes of deaths (number of deaths, CSMFs (%)), by Female

Bangladesh	No	%	Myanmar	No	%	Thailand	No	%	Lao PDR	No	%	Cambodia	No	%
Cerebrovascular	29	14	Cerebrovascular	24	21	Neoplasms	71	20	Digestive related	72	12	Neoplasms	21	24
Chronic respiratory diseases	29	14	Digestive related	16	14	Cardiovascular	47	13	Neoplasms	58	10	Digestive related	17	19
Neoplasms	27	13	Neoplasms	16	14	Accidents and injuries	44	12	Diabetes	58	10	Cardiovascular	10	11
Cardiovascular	26	12	Accidents and injuries	8	7	Genito-urinary related	34	9	Cardiovascular	42	7	Cerebrovascular	7	8
Diarrhoeal related	16	8	Chronic respiratory diseases	7	6	Cerebrovascular	32	9	Genito-urinary	38	6	Diabetes	5	6
Genito-urinary related	13	6	Cardiovascular	6	5	Chronic respiratory diseases	27	8	Pneumonia	36	6	Tuberculosis	4	5
Accidents & Injuries	12	6	Genito-urinary related	5	4	Digestive related	18	5	cerebrovascular	26	4	Accidents and injuries	4	5
Tuberculosis	8	4	Tuberculosis	3	3	Sepsis	17	5	Tuberculosis	23	4	Genito-urinary	3	3
Maternal conditions	7	3	Diarrhoeal related	2	2	Covid	11	3	Accidents and injuries	20	3	Diarrhoeal related	2	2

Pneumonia	5	2	Maternal	2	2	Neuro-psychiatric conditions	10	3	chronic respiratory diseases	20	3	Chronic respiratory/pneumonia	1	1

Appendix 11 Crude death rate per 1,000 population, by country

Countries	Bangladesh	Myanmar	Thailand	Lao PDR	Cambodia
Crude death rate	5	19	8	9	3

Appendix 12 Age and YLL distribution by major cause groups

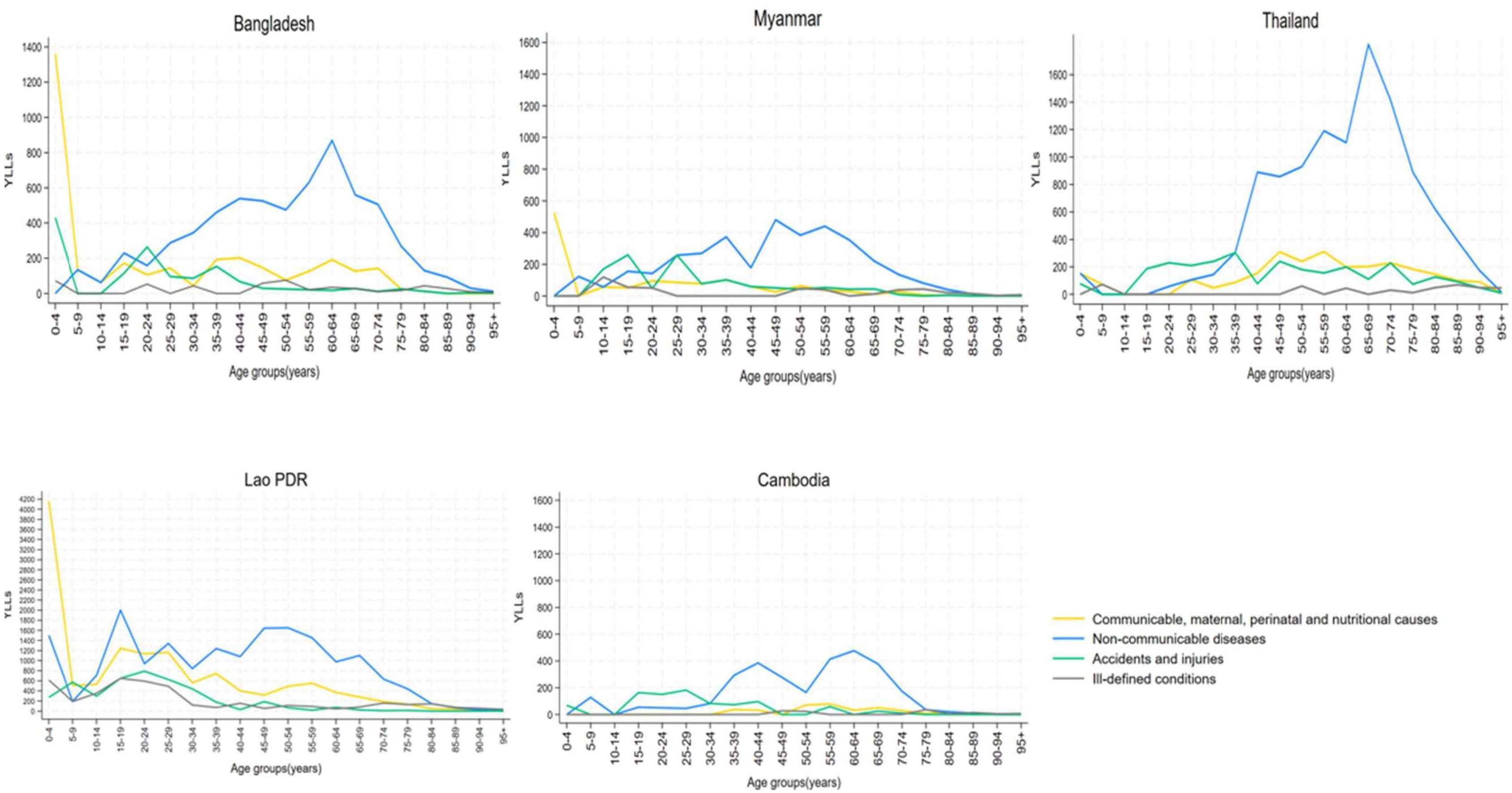

Appendix 13 Discrepancy, reconciliation, and adjudication rates across the sites

Study sites	Initial discrepancy rate (%)	Reconciliation rate (%)	Adjudication rate (%)
Bangladesh	30	87	13
Myanmar	21	90	10
Thailand	23	85	15
Lao PDR	27	88	12
Cambodia	15	90	10

Initial discrepancy rate: The proportion of cases with different cause-of-death assignments by the two independent coders at first assessment.
Reconciliation rate: The proportion of initially discrepant cases that were resolved by consensus between the two coders.
Adjudication rate: The proportion of initially discrepant cases that required resolution by a third senior coder after reconciliation.
